# Single-cell deconvolution reveals high lineage- and location-dependent heterogeneity in mesenchymal multivisceral stage 4 colorectal cancer

**DOI:** 10.1172/JCI169576

**Published:** 2023-12-28

**Authors:** Christopher Berlin, Bernhard Mauerer, Pierre Cauchy, Jost Luenstedt, Roman Sankowski, Lisa Marx, Reinhild Feuerstein, Luisa Schaefer, Florian R. Greten, Marina Pesic, Olaf Groß, Marco Prinz, Naomi Ruehl, Laura Miketiuk, Dominik Jauch, Claudia Laessle, Andreas Jud, Esther A. Biesel, Hannes Neeff, Stefan Fichtner-Feigl, Philipp A. Holzner, Rebecca Kesselring

**Affiliations:** 1 Department of General and Visceral Surgery, Faculty of Medicine, University of Freiburg, Freiburg, Germany.; 2 German Cancer Consortium (DKTK) Partner Site, Freiburg, Germany.; 3 German Cancer Research Center (DKFZ), Heidelberg, Germany.; 4 IMM-PACT Clinician Scientist Program,; 5 Institute of Neuropathology,; 6 Single-Cell Omics Platform Freiburg, Faculty of Medicine, University of Freiburg, Freiburg, Germany.; 7 Institute for Tumor Biology and Experimental Therapy, Georg-Speyer-Haus, Frankfurt/Main, Germany.; 8 Signalling Research Centres BIOSS and CIBSS,; 9 Center for Basics in NeuroModulation (NeuroModulBasics), Faculty of Medicine, and; 10 EXCEL Excellent Clinician Scientist Program, Faculty of Medicine, University of Freiburg, Freiburg, Germany.

**Keywords:** Cell biology, Oncology, Cancer immunotherapy, Colorectal cancer, Mouse models

## Abstract

Metastasized colorectal cancer (CRC) is associated with a poor prognosis and rapid disease progression. Besides hepatic metastasis, peritoneal carcinomatosis is the major cause of death in Union for International Cancer Control (UICC) stage IV CRC patients. Insights into differential site-specific reconstitution of tumor cells and the corresponding tumor microenvironment are still missing. Here, we analyzed the transcriptome of single cells derived from murine multivisceral CRC and delineated the intermetastatic cellular heterogeneity regarding tumor epithelium, stroma, and immune cells. Interestingly, we found an intercellular site-specific network of cancer-associated fibroblasts and tumor epithelium during peritoneal metastasis as well as an autologous feed-forward loop in cancer stem cells. We furthermore deciphered a metastatic dysfunctional adaptive immunity by a loss of B cell–dependent antigen presentation and consecutive effector T cell exhaustion. Furthermore, we demonstrated major similarities of this murine metastatic CRC model with human disease and — based on the results of our analysis — provided an auspicious site-specific immunomodulatory treatment approach for stage IV CRC by intraperitoneal checkpoint inhibition.

## Abstract 2

Multivisceral stage IV colorectal cancer shows a high lineage-dependent and location-dependent heterogeneity that necessitates site-specific therapy in a mouse model.

## Graphical abstract

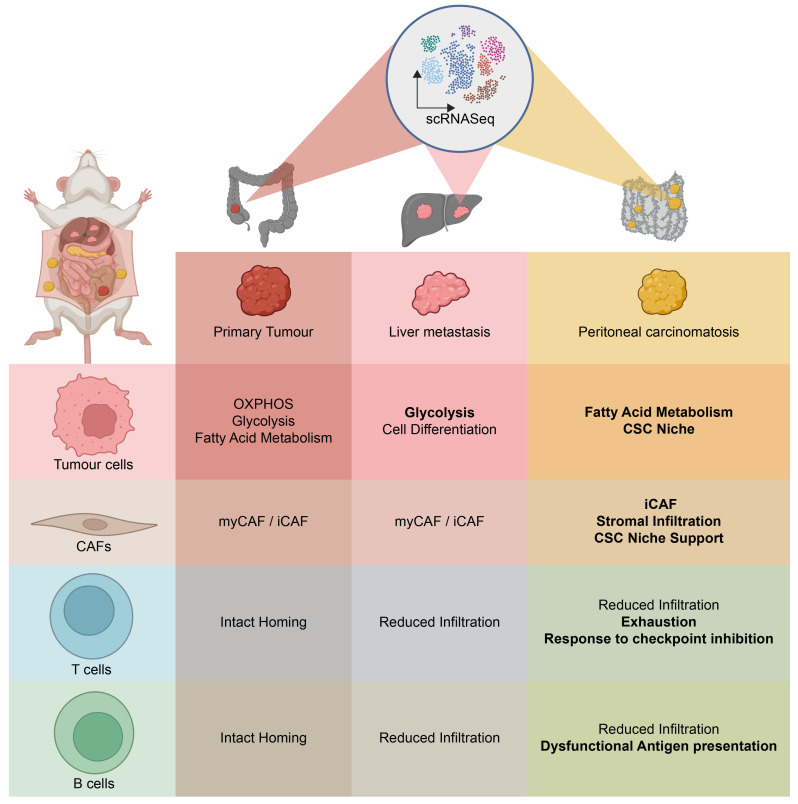

## Introduction

Colorectal cancer (CRC) is the second most diagnosed cancer in females and the third most common in males, with about 0.8 million deaths worldwide per year (1). Whereas primary CRC in early stages is mostly curable by surgery, mortality rates of metastatic cancer are still high (2). Therefore, it is critical to understand the entire multistep process of metastasis, also considering a strong intermetastatic heterogeneity between tumor locations, accounting for tumor-specific adaptations to local environments. While an immunosuppressive phenotype of the tumor microenvironment (TME) is already described for colorectal liver metastases (CRLM) (3–6), the intermetastatic differential reconstitution of the TME in peritoneal carcinomatosis (PC) has not been investigated yet, since appropriate mouse models mimicking human metastasis are still missing (7–11). Due to limitations of treatment options, PC is associated with the worst survival in CRC patients and occurs most frequently in a mesenchymal subtype of CRC belonging to the consensus molecular subtype 4 (CMS4) subtype (12, 13). This molecular subtype is characterized by the poorest survival rate, represents approximately one-fourth of CRC patients (14), and shows an immune-tolerant, inflamed TME (15). Hence, there is an urgent need to understand the molecular and cellular landscape during CRC metastasis to different metastatic locations without the influence of intermittent therapeutic interventions.

Here, we aim to understand the site-specific landscape of metastasized CRC by single-cell RNA-Seq (scRNA-Seq) using a surgical tumor organoid–driven orthotopic CRC mouse model. Our analyses unravelled location-dependent epithelial, stromal, and immunological changes during metastasis to the liver and the peritoneum. While we observed a self-sustaining metastatic stem cell niche in PC, which was furthermore supported by inflammatory fibroblasts, our data suggested an impaired antitumoral adaptive immunity during metastasis with a conversely enhanced effector function of innate immune cells. Our findings confirmed the proposed mouse model as a state-of-the-art model for studying the molecular and cellular landscape of multivisceral CRC, resembling human metastasis. Additionally, we identified individual cellular compartments of the TME as promising therapeutic targets, and we suggest a necessity for a site-specific adaptation of therapeutic approaches.

## Results

### A distinct cellular and functional landscape of murine primary CRC, liver metastases, and PC.

**Figure 1:**
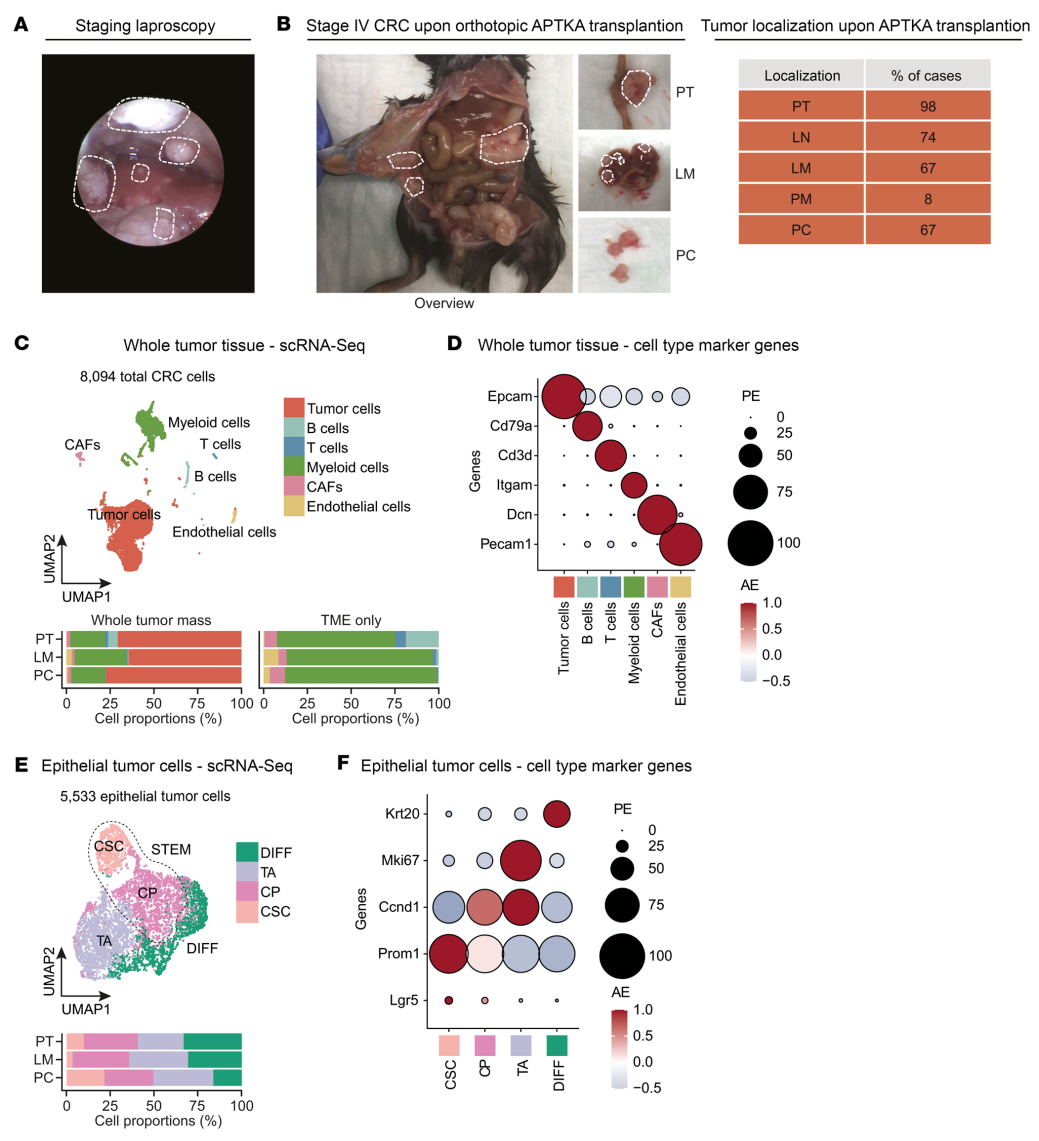
A distinct cellular and functional landscape of murine primary CRC, LM, and PC. (**A**) Exemplary screenshot during murine laparoscopy after 8 weeks. White dashed lines mark interenteric and abdominal wall peritoneal tumors as well as LMs. (**B**) Overview of murine metastasized stage IV CRC upon animal sacrifice (left panel). Quantification of location-dependent macroscopic tumor mass as percentages. Locations: PT, LN, LM, PM, and PC (right panel). (**C**) UMAP plot of 8,094 cells identified by joint application of RCA and CCA and color coded by cell type (upper panel). Proportions of all cell types in PT, LM, and PC (left lower panel) or of the TME (right lower panel) on average are shown. (**D**) Canonical marker gene expression for multiple cell types centered to the average expression of each gene across all cells. The dot size represents the proportion of expressing cells in each cluster. PE, percentage expressed; AE, average expression. (**E**) UMAP plot of 5,533 tumor cells identified by joint application of RCA and CCA and color coded by cell subtype (upper panel). Proportions of all cell types in PT, LM, and PC (lower panel) on average are shown. (**F**) Canonical marker gene expression for epithelial cell subtypes centered to the average expression of each gene across all cells. Dot size represents the proportion of expressing cells in each cluster.

**Figure 2:**
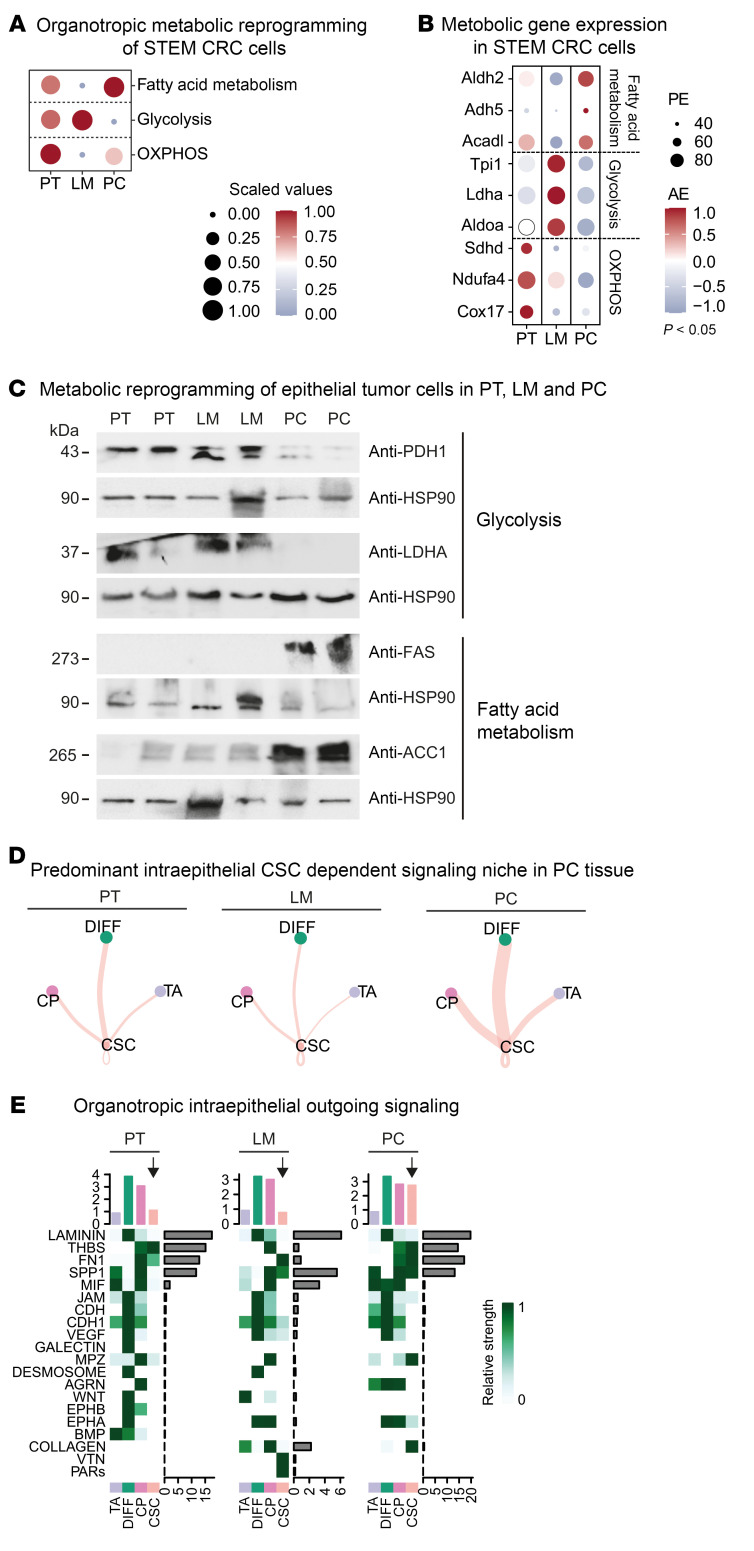
Location-specific metabolic reprogramming of the STEM in multivisceral CRC. (**A**) Metabolic activity analysis in the STEM of PT, LM, and PC. Circle size and color represent scaled metabolic score. (**B**) Expression of the top 3 differentially expressed metabolic genes in the STEM of PT, LM, and PC centered to the average expression of each gene across all locations. Dot size represents the proportion of expressing cells in each cluster. *P* < 0.05. (**C**) Representative Western blot showing expression of depicted proteins in pooled epithelial lysates (*n* = 5 animals per column) from murine PT, LM, and PC. Separate loading control for each Western blot: HSP90. The experiment was performed twice. (**D**) Significant L-R pairs between any pair of 2 epithelial cell populations in PT, LM, and PC. Width represents communication probability. (**E**) Heatmap shows the relative importance in depicted signaling pathways for each cell group based on the computed centrality measures in PT, LM, and PC. Arrows indicate outgoing signaling patterns from CSCs.

To set up a mouse model for the investigation of treatment-naive hepatic and peritoneal metastasis of CRC, we adapted the surgical orthotopic transplantation model from Fumagalli et al. (10, 11) and implanted murine organoids, which are deficient for *Apc*, *Tp53*, and *Tgfbr2* and carry a *Kras*G12D mutation and an activated/myristoylated isoform of AKT1 (hereafter termed APTKA) (16, 17) under the subserosa of the cecum of immunocompetent C57BL/6J mice (Supplemental Figure 1, A–C, and Supplemental Video 1; supplemental material available online with this article; https://doi.org/10.1172/JCI169576DS1). Invasive primary tumor (PT) growth as well as hepatic and peritoneal spread were monitored by video-guided laparoscopy (Figure 1A and Supplemental Video 2), and peritoneal tumor load was quantified using an adapted PC index (PCI) scoring system (Supplemental Figure 1, D and E). Upon animal sacrifice, primary CRC (PT) growth rate was 98%. Liver metastasis (LM) and pulmonary metastasis (PM) rates were 67% and 8%, respectively. Lymph node metastases (LN) occurred in 74% of all cases. Ultimately, a high percentage of animals (67 %) developed PC (Figure 1B). Gene-expression profiling indicated that the PT tissue resembled human mesenchymal CRC (CMS4) (Supplemental Figure 1F), which is shown to frequently metastasize to the peritoneal cavity when being compared with other CMSs (13, 18). To elucidate the cellular landscape of multivisceral CRC, we analyzed 8,094 cells (Supplemental Figure 1, G and H) from whole-tumor samples of PT, LM, and PC by droplet-based scRNA-Seq. We used unsupervised clustering and 2D embedding using uniform manifold approximation and projection (UMAP) as well as a marker-based annotation strategy to identify 6 major cell types: tumor cells (*Epcam^+^*), B cells (*Cd79a^+^*), T cells (*Cd3d^+^*), myeloid cells (*Itgam^+^*), cancer associated fibroblasts (CAFs) (*Dcn^+^*), and endothelial cells (*Pecam1^+^*) (Figure 1, C and D). Interestingly, cellular compositions of metastatic tissues (LM and PC) differed substantially from those of PT: quantification of populations in the TME revealed a differing immunological TME in both metastatic CRC tissues, with increased myeloid cell populations in LM and PC as well as an obvious decrease of B cell and T cell counts in LM and a nearly complete exclusion of both immune cell populations in PC. Meanwhile, CAFs were most strongly represented in PC and increased endothelial cell populations were found in both metastatic sites with the highest abundance in LM tissue (Figure 1C and Supplemental Figure 1H). CD45 and PDGFRB IHC further confirmed the reduction of immune cell infiltration in metastatic tissue and the reciprocal stromal increase in PC (Supplemental Figure 1I). Concerning the epithelial compartment, we further identified 4 epithelial subpopulations in 5,533 tumor cells: cancer stem cells (CSCs) (*Lgr5*^+^ and *Prom1*^+^) and cycling progenitor cells (CPs) (*Prom1*^+^ and *Ccnd1*^+^), summarized as stem cell compartment (STEM), since CP still expressed stem cell marker genes. A highly proliferative cellular compartment was annotated as transient amplifying cells (TA) (*Mki67*^+^). Finally, differentiated cells (DIFFs) showed high expression of *Krt20* (Figure 1, E and F). Of note, the distribution pattern of these epithelial subpopulations was tumor location specific: while the DIFF population was the largest population in PT, the proliferating TA populations dominated in both metastatic sites. Most strikingly, we found a massive deregulation of the CSC compartment upon CRC metastasis: while being reduced to approximately one-third in LM compared with PT, the CSC population was increased more than2-fold in PC (Figure 1E). Based on this observation, we questioned whether the epithelial CMS (intrinsic CMS/iCMS) based on the framework established by Joanito et al. (19) was location dependent in our multivisceral CRC model. Thus, we formulated gene scores for iCMS2 (327 genes; iCMS2 score up) and iCMS3 (67 genes; iCMS3 score up) and observed distinct patterns of gene-score expression across all 3 locations. Specifically, iCMS2-associated genes displayed enhanced expression in PT and PC, while iCMS3-associated transcripts exhibited upregulation within the LM epithelial compartment. Consequently, we have designated the PT and PC epithelium as iCMS2, whereas the LM epithelium has been categorized as iCMS3, emphasizing the niche-specific reprogramming of the epithelium during metastasis (Supplemental Figure 1G). To further understand the mechanism of the potent STEM niche, especially during metastasis, we analyzed the metabolic scores of the STEM at each site. Strikingly, VISION-based single-cell metabolic analysis (20) revealed a distinct phenotype of especially LM and PC STEM cells: while STEM cells in PT partially obtained scores for fatty acid degradation, glycolysis/ gluconeogenesis, and predominantly oxidative phosphorylation (OXPHOS), the respective cellular compartment in metastases was metabolically reprogrammed in a distinct manner. While LM STEM showed the highest scores for glycolysis/gluconeogenesis, PC STEM was characterized by high metabolic scores of fatty acid degradation (Figure 2A). In line with this, analysis of representative genes for each metabolic pathway showed equivalent differential location-dependent expression (Figure 2B). To further substantiate this metabolic deregulation in the STEM, we purified epithelial cells from all 3 sites and confirmed location-specific deregulations of metabolic patterns of glycolysis and fatty acid metabolism on protein levels in the epithelium: epithelial cells from LM show elevated levels of proteins involved in glycolytic activity, while key enzymes of fatty acid metabolism were overexpressed in PC tumor cells (Figure 2C). Importantly, lipid metabolism is already known to be associated with CSC maintenance (21). To infer a potential successive niche that promotes the intercellular and CSC-specific crosstalk in the epithelial niche, we computed cell-cell interactions using a ligand-receptor–dependent (L-R–dependent) algorithm (22). Consistently, we found increased outgoing signaling from the CSC compartment toward all epithelial subtypes in PC (Figure 2D). Signaling pathway analyses of respective L-R pairs showed increased outgoing collagen, osteopontin (SPP1), fibronectin, and thrombospondin signaling from CSCs in PC (Figure 2E and Supplemental Figure 2, A and B), which are already known to be associated with dedifferentiation, stem cell support, and metastasis (23–28). Collectively, our data demonstrate an intersite heterogenic reorganization of the cellular composition at respective PT and metastases sites. Additionally, we were able to decipher a site-specific iCMS of tumor epithelium and metabolic reprogramming of the stem cell compartment, leading to a self-supportive feed-forward niche in the epithelial compartment of treatment-naive murine PC. These data implied a site-specific environmental adaption during multivisceral metastasis of mesenchymal CRC.

### Intermetastatic differences in stromal cell dynamics during CRC metastasis.

**Figure 3:**
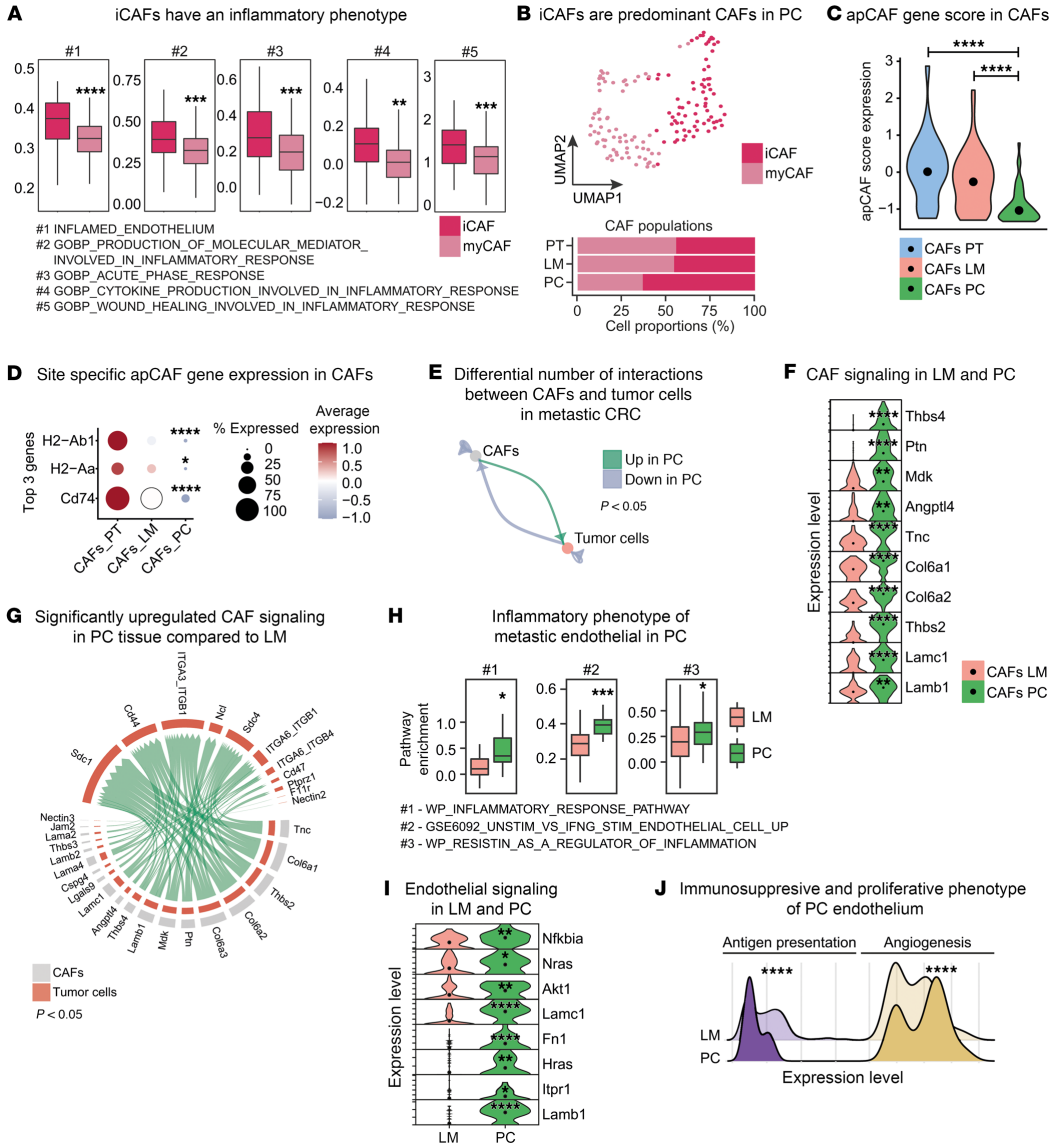
Intermetastatic differences in stromal cell dynamics during CRC metastasis. (**A**) Pathway enrichment analysis with published inflammatory signatures in myCAFs and iCAFs. (**B**) UMAP plot of myCAFs and iCAFs identified by joint application of RCA and CCA and color coded by cell subtype (upper panel). Proportions of cell subtypes in PT, LM, and PC tissue (lower panel) on average are shown. (**C**) Expression level of apCAF gene score (Supplemental Table 4) in CAFs from PT, LM, and PC depicted as violin plot. (**D**) Expression of the top 3 differentially expressed genes involved in antigen presentation in CAFs of PT, LM, and PC centered to the average expression of each gene across all locations. Dot size represents the proportion of expressing cells in each cluster. (**E**) Differential numbers of L-R interactions between CAFs and tumor cells in LM compared with PC. Green, upregulation in PC compared with LM; blue, downregulation in PC compared with LM. (**F**) Expression level of significantly upregulated exemplary L or R genes in PC compared with LM depicted as stacked violin plot. (**G**) Significantly upregulated L-R pairs between CAFs and tumor cells in PC compared with LM depicted as circle plot. (**H**) Pathway enrichment analysis in endothelial cells from LM and PC with published gene signatures. (**I**) Expression levels of significantly upregulated exemplary genes involved in inflammation and proliferation in PC and LM depicted as stacked violin plot. (**J**) Expression levels of antigen presentation and angiogenesis gene scores (Supplemental Table 4) in endothelial cells from LM and PC depicted as ridge plot. *P < 0.05; **P < 0.01; ***P < 0.001; ****P < 0.0001, Student’s *t* test.

CAF polarization to an inflammatory phenotype (iCAF) in the TME of CRC is already known to be prognostically relevant, to drive therapy resistance and metastasis by supporting immune evasion, and to promote invasive capacity of tumor cells (16, 29–31). We further subclustered CAFs in our murine data set into 2 clusters (Supplemental Figure 3A). Gene set enrichment analysis (GSEA) indicated upregulation of genes involved in inflammatory pathways in cluster 2 in comparison with cluster 1. Accordingly, cells in cluster 2 were classified as iCAFs and cells in cluster 1 were termed myofibroblasts (myCAF) (Figure 3A). Site-specific quantification of respective cell populations revealed predominant existence of iCAFs in PC (Figure 3B). Reciprocally to the upregulation of iCAF gene expression, we found decreased expression of genes characterizing antigen-presenting CAFs (apCAFs) in PC (Figure 3C), which are reported to be involved in homing and activation of immune cells in the TME of solid tumors (32, 33), e.g., *H2-Ab1*, *H2-Aa*, and *Cd74* (Figure 3D). Accordingly, we have already shown that B cell and T cell populations were strongly reduced in murine PC (Figure 1C).

To understand the site-specific heterogeneity of a CAF-specific intercellular communication, especially in metastatic CRC, we inferred communication probabilities between CAFs and tumor cells in LM and PC. Of note, we found increased numbers of putative interactions between both cell populations in PC and identified CAFs in PC as predominant sender population (Figure 3E). We then identified increased expression of genes, e.g., *Thbs4*, *Ptn*, *Mdk*, *Angptl4*, *Tnc*, *Col6a1*, *Col6a2*, *Thbs2*, *Lamc*, and *Lamb* in CAFs from PC (Figure 3F). Differential coexpression analyses showed overexpression of respective L-R pairs between CAFs and tumor cells in PC, suggesting increased cell-cell communication (Figure 3G and Supplemental Figure 3B). L-R pairs, e.g., *Lamb1/Cd44*, are already known to drive the metastatic capacity of CRC and to support intratumoral CSC function by upregulation of stem cell–supporting pathways (34, 35). Considering this site-specific reprogramming of CAFs in accordance with the elevated numbers of CSCs in PC (Figure 3D), we further analyzed the differentially expressed genes between CSCs from PC and LM to understand a potential CAF-mediated transcriptomic reprogramming of this specific epithelial cell population. Unbiased GSEA revealed significant upregulation of Kyoto Encyclopedia of Genes and Genomes (KEGG) Gene Ontology (GO) terms associated with stem cell support, such as Wnt and Hippo signaling (Supplemental Figure 3C). These data suggest that CAFs in PC support the epithelial niche by directly supporting CSC survival. Furthermore, endothelial cells of the tumor stroma are already used as therapeutic targets (36). To understand the site-specific function in metastatic CRC, metastatic endothelial cells were conducted to GSEA, which confirmed an inflammatory phenotype in PC endothelium compared with LM (Figure 3H). Accordingly, differential expression analysis showed increased expression of genes, e.g., *Lamb1*, *Itpr1*, *Hras*, *Fn1*, *Lamc1*, *Akt1*, *Nras*, and *Nfkbia*, in PC (Figure 3I). Accordingly, with the observed upregulation of genes involved in inflammation and proliferation, we set up gene scores for antigen presentation and angiogenesis with established gene panels (Supplemental Table 4). Interestingly, we obtained a loss of antigen-presentation capacity paired with an increased angiogenic function in PC endothelium (Figure 3J), suggesting a locoregional attenuated immune stimulation and increased neovascularization supporting the outgrowth of PC. Taken together, our data show a distinct and PC-specific reprogramming of the metastatic stromal cells. We show increased support of the locoregional CSC niche by CAFs as well as enhanced immunosuppressive inflammation and angiogenic activity of endothelial cells.

### Location-specific antitumoral immunity during CRC disease progression.

Having shown reduction of immune cells of adaptive immunity during metastasis to the peritoneal cavity, we aimed to describe the innate immune cell compartment in multivisceral CRC. Whereas myeloid cells in CRLM have already been investigated (6), understanding of the role of myeloid cells in PC is still lacking. Therefore, we analyzed *Itgam*^+^ myeloid cells from PT, LM, and PC (Supplemental Figure 4A). Quantification of subtypes revealed a decrease of potentially antitumoral conventional dendritic cells (cDCs) during disease progression with a concomitant increase of proliferative (*Mki67*^+^) macrophages. Inflamed tissue macrophages and neutrophils could only be identified in LM. Moreover, we analyzed the location-dependent reprogramming of *Mrc1*^+^ macrophages and found concordant polarization with already published data (6). In metastases, *Mrc1*^+^ macrophages exhibited upregulation of genes involved in M2 polarization and immunosuppression, such as *Lipa*, *Marco*, and *Id3*, while *Mrc1*^+^ macrophages from PT expressed higher levels of inflammatory cytokines, such as, e.g., *Tnf*, *Ccl3*, *Ccl4*, *Il23a*, and *Cxcl1* (Supplemental Figure 4B).

**Figure 4:**
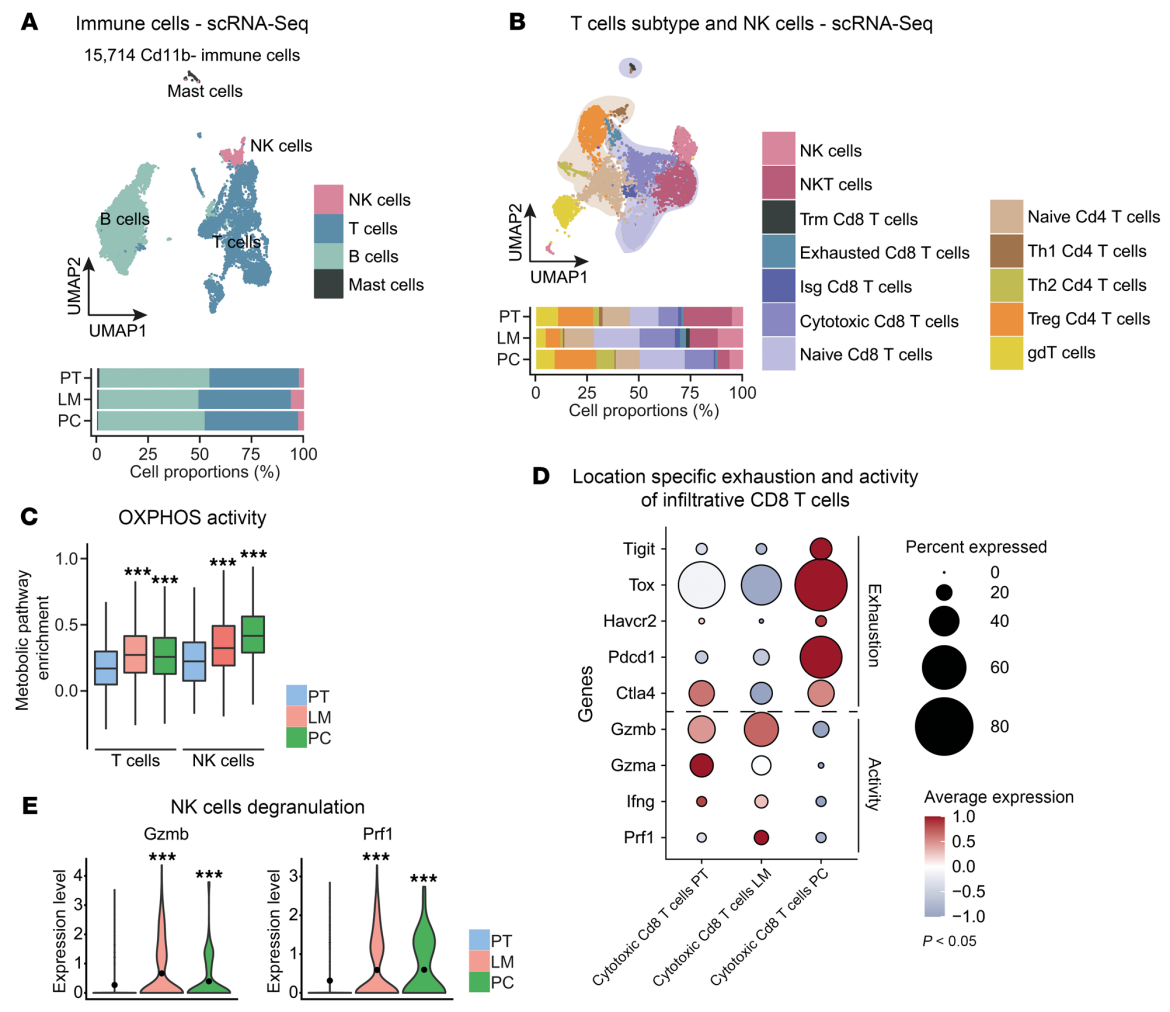
Location-specific antitumoral immunity during CRC metastasis. (**A**) UMAP plot of 15,714 CD45^+^CD11b^–^ immune cells identified by joint application of RCA and CCA and color coded by cell subtype (upper panel). Proportions of all cell types in PT, LM, and PC (lower panel) on average are shown. (**B**) UMAP plot of T and NK cells identified by joint application of RCA and CCA and color coded by cell type (upper panel). Proportions of all cell types in PT, LM, and PC (lower panel) on average are shown. (**C**) Metabolic activity analysis in T cells and NK cells of PT, LM, and PC. Metabolic score depicted as box plot. (**D**) Expression of significantly differentiated genes involved in activity and exhaustion of cytotoxic CD8^+^ T cells from PT, LM, and PC centered to the average expression of each gene across all locations. Dot size represents the proportion of expressing cells in each cluster. *P* < 0.05. (**E**) Expression level of Gzmb and Prf1 in NK cells from PT, LM, and PC depicted as violin plot. ***P < 0.001, Student’s *t* test.

Since whole-tumor data showed a massive decrease of B and T cells during metastasis (LM and PC) (Figure 1C), we purified CD45^+^CD11b^–^ infiltrative cells from all 3 sites by FACS (Supplemental Figure 4C) to enable analysis of the adaptive compartments. Here, we analyzed 15,714 *Cd79a*^+^ B cells, *Cd3d*^+^ T cells, *Klrb1*^+^ NK cells, and *Kit*^+^ mast cells by scRNA-Seq after quality control (Supplemental Figure 4, D and E). Annotation of cells was performed using a marker-based strategy (Supplemental Figure 4F and Figure 4A). Quantification of cell types revealed low abundance of mast cells with a predominant existence in PT, while we obtained approximately equal location-dependent compartment sizes for B and T cells as well as a predominance of NK cells in LM (Figure 4A). Analyzing these immune cell clusters in detail, T cells contained multiple clusters representing the transition from naive to effector CD4^+^, CD8^+^, and γδ T cells, with the latter decreased in LM (Supplemental Figure 4G). Further subcluster analyses from T cells marked increased infiltration of naive CD8^+^ T cells, exhausted CD8^+^ T cells, *Foxp3*^+^ Tregs, and cytotoxic CD8^+^ T cells during metastasis (Figure 4B). To molecularly understand a potential functional reprogramming of T and NK cell subtypes, we performed VISION-based metabolic scoring on the T and NK cell populations. OXPHOS can be associated with terminal T cell exhaustion and dysfunction in cancer (37–39), while it metabolically fosters antitumoral NK T cell and NK cell activity (40, 41). Accordingly, highest baseline OXPHOS scores were witnessed in exhausted CD8^+^ T cells (Supplemental Figure 4H), and location-dependent analysis of OXPHOS activity revealed increased scoring in T and NK cells of both metastatic locations compared with PT (Figure 4C). Additionally, this metabolic conversion was also obtained in metastatic T cells on a subtype level (Supplemental Figure 4I). While the number of potentially antitumoral adaptive immune cells such as cytotoxic CD8^+^ T cells increased in metastases, we found upregulation of markers for T cell exhaustion and downregulation of markers for T cell activation in PC (Figure 4D). This observation was in accordance with elevated OXPHOS genes in PC. Furthermore, we also analyzed markers for degranulation in NK cells, since we observed elevated numbers of this population in metastases. Interestingly, the degranulation markers *Gzmb* and *Prf1* were expressed in a higher manner in metastases, suggesting increased cytotoxicity (Figure 4E). These data depict a dysfunctional phenotype and metabolic reprogramming of adaptive antitumoral immune cells in LM and PC as well as an increased cytotoxic function of infiltrative NK cells in respective lesions.

### Impairment of B cell networks and altered adaptive immunity in metastasized CRC.

**Figure 5:**
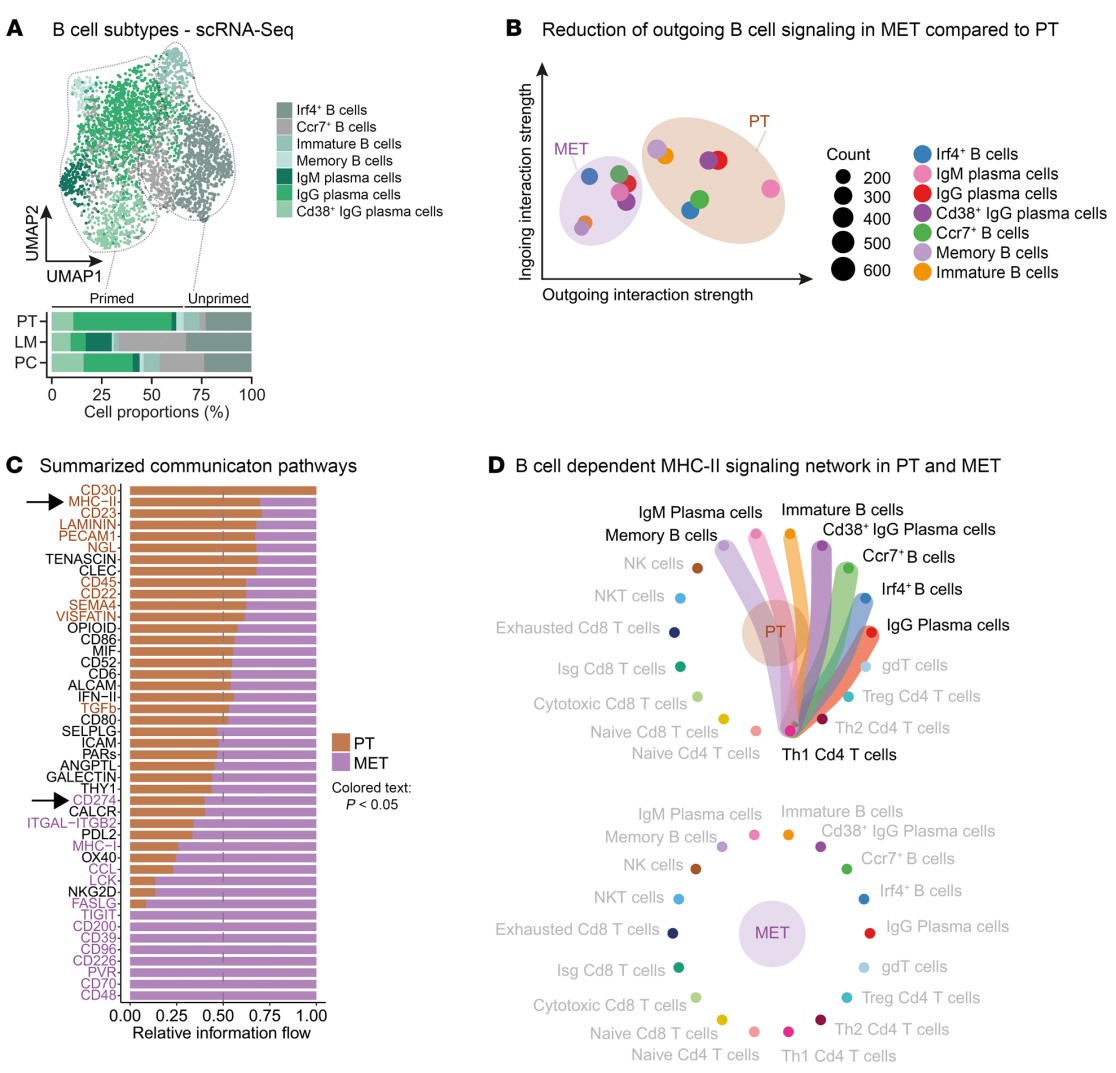
Impairment of B cell networks in metastasized CRC. (**A**) UMAP plot of 3,355 B cells identified by joint application of RCA and CCA and color coded by cell subtype (upper panel). Proportions of all cell subtypes in PT, LM, and PC (lower panel) on average are shown. Dashed lines mark primed and unprimed/immature B cell subtypes. (**B**) Ingoing and outgoing interaction strength of B cells with joint projection and clustering B cell subtypes onto shared 2D manifold according to their local descent. MET, combined analysis for LM and PC. Circle or square size is proportional to the signaling strength of respective cellular subtype. Different colors represent different B cell subtypes. (**C**) Unbiased overall information flow of signaling networks by summarizing all the communication probabilities in respective networks. All the significant signaling pathways were ranked based on their differences of overall information flow within the inferred networks between PT and MET. Brown, enriched in PT; purple, enriched in MET. (**D**) Significant L-R pairs of the MHC-II signaling network between B cell subtypes and Th1 Cd4^+^ T cells in PT and MET. Edge width represents MHC-II–dependent communication probability.

Precondition for adequate adaptive cytotoxicity is a subtle concerted interplay of T and immunoregulatory B cells that participate in antibody production and antigen presentation (42–44). To analyze B cell functionality and T and B cell interactions in the distinct locations, we further subclustered 3,355 B cells. We identified 7 B cell subtypes incorporating different developmental stages: immature *Cd24a*^+^ B cells, *Irf4*^+^ B cells, and naive *Ccr7*^+^ B cells and populations during stages of B cell maturation or unprimed B cells (including memory B cells, IgM and IgG plasma cells, and CD38^+^ IgG plasma cells). Memory B cells, IgM^+^ plasma cells, IgG^+^ plasma cells, and *Cd38*^+^ IgG^+^ plasma cells typified B cell subtypes that already were primed upon antigen recognition (Figure 5A and Supplemental Figure 5A). We found unequal distribution of B cell subtypes in primary and metastases: while primed B cell subtypes were predominantly occurring in PT, most immature and naive subtypes were found in LM and PC (Figure 5A). To decipher the potential antigen-presenting function of B cells, we analyzed differential L-R signaling interactions in B and T cell populations and found B–T cell as well as inter–T cell interactions as expected (Supplemental Figure 5B). To compare the outgoing B cell signaling between PT and metastatic tissue, LM and PC samples were pooled and termed as MET. Strikingly, we obtained a reduction of outgoing B cell signaling in all MET B cell subtypes when being compared with PT B cells, suggesting an altered B cell function specifically in metastases (Figure 5B). Summarized differential pathway analysis confirmed downregulation of MHC-II signaling pathways in MET B and T cells, implicating a reduction of MHC-II–dependent antigen presentation, accompanied by increased PD/L1 pathway activation (Figure 5C). We assumed a reduction of the canonical MHC-II–dependent Th1 CD4^+^ T cell interaction and could show a drastic loss of MHC-II–driven L-R pairs between B and Th1 CD4^+^ T cells in MET (Figure 5D). Additionally, we found increased mRNA expression of *Cd274* (encoding for PD-L1) in MET Th2 CD4^+^ T cells, which could indicate a worsened immunosuppressive phenotype of T cells in metastases (Supplemental Figure 5C).

**Figure 6:**
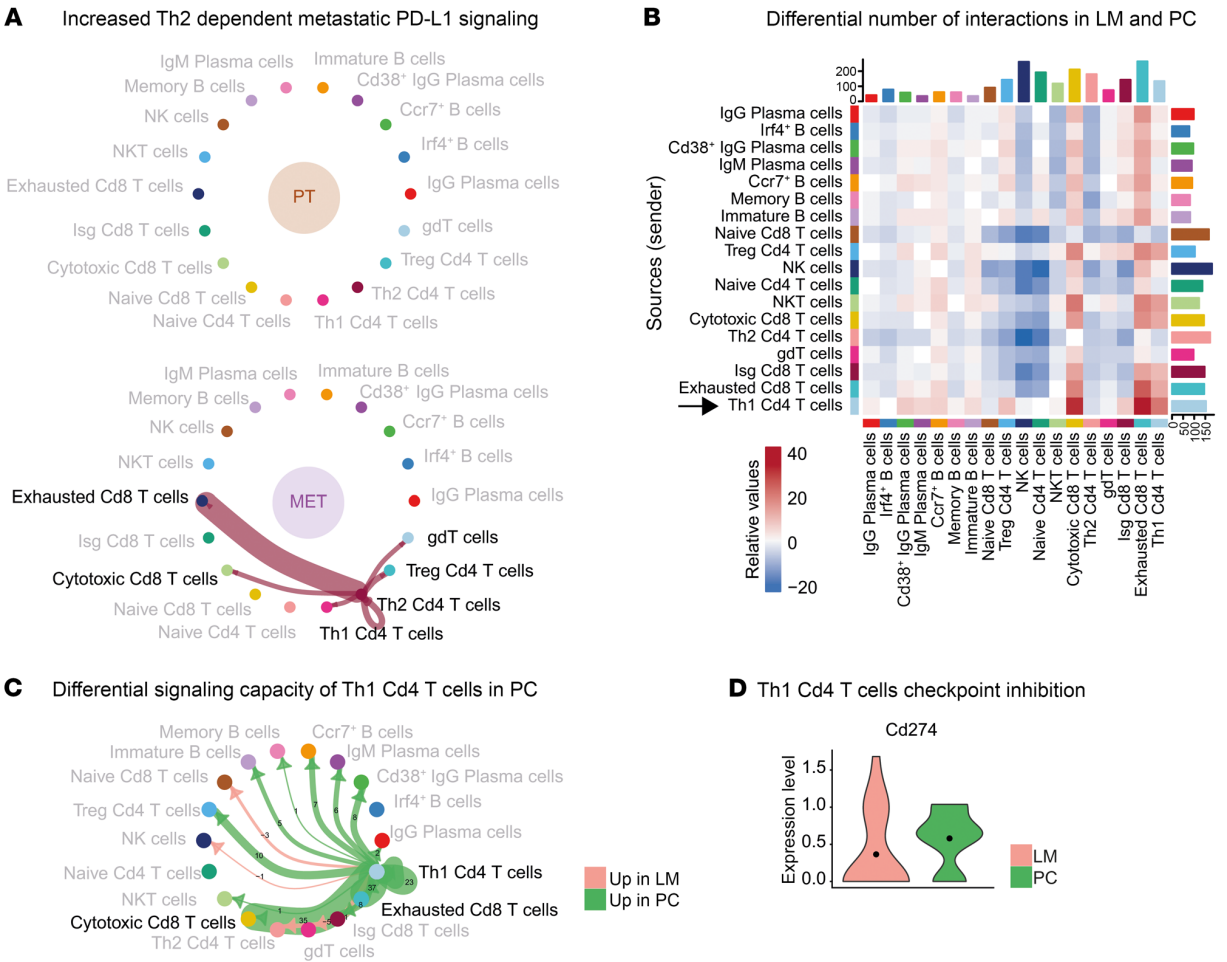
Intermetastatic alterations of adaptive immune responses to multivisceral CRC. (**A**) Significant L-R pairs of the PD-L1 signaling network between Th2 Cd4^+^ T cells and other T cell subtypes in PT and MET. Edge width represents MHC-II–dependent communication probability. (**B**) Heatmap depicting the differential number of interactions between B and T cell subtypes in LM and PC. Colors represent relative values. (**C**) Differential numbers of L-R interactions between Th1 Cd4^+^ T cells and B cell and T cell subtypes in LM compared with PC. Green, upregulation in PC compared with LM; red, upregulation in LM compared with PC. Numbers indicate differentially regulated L-R pairs. (**D**) Expression levels of *Cd274* in PC compared with LM depicted as violin plot.

To further uncover intermetastatic differences in the intercellular regulation of effector T cell function, we also analyzed differential signaling sender capacities of B and T cells between LM and PC (Figure 6A). Here, we identified Th1 CD4^+^ T cells as prominent sender cells, especially toward cytotoxic and exhausted CD8^+^ T cells (Figure 6B), with increased sender capacities in PC (Figure 6C). PD-L1 signaling was significantly upregulated in the summarized communication pathway analysis in PC (Supplemental Figure 6A), and Th1 CD4^+^ T cells from PC expressed higher mRNA levels of *Cd274* (Figure 6D). Consequently, further differential Th1 Cd4^+^ T cell–dependent PD-L1 signaling analysis showed a PC-specific increase of PD-L1–dependent signaling toward effector T cells (Supplemental Figure 6B). Taken together, these data imply a potentially disrupted antigen-presenting function of B cells represented by a loss of MHC-II–dependent L-R signaling in metastases toward T cells. Furthermore, we demonstrate a location-specific PD-L1–dominant Th1 CD4^+^ T cell phenotype, with increased PD-L1 L-R interaction toward cytotoxic CD8^+^ T cells in PC. These results underline the establishment of site-specific TMEs with altered adaptive immune responses.

### Murine multivisceral APTKA CRC mimics human stage IV CRC.

**Figure 7:**
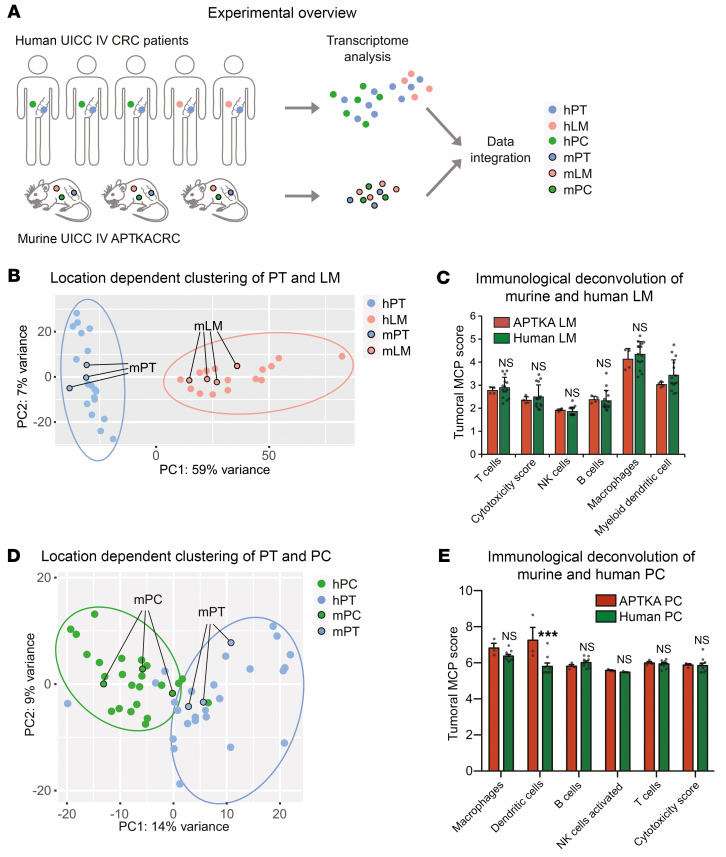
Murine multivisceral APTKA CRC mimics human stage IV CRC. (**A**) Experimental overview for integrated comparison of human and murine RNA-Seq data from PT and PC. (**B**) Batch-corrected PCA of 15 patient-matched hPT/hLM samples and 3 mPT/4 mLM samples shows location-dependent clustering of human and murine transcriptomes. (**C**) Estimated MCP scores reflecting immune cell infiltration in RNA samples from murine and human LM. (**D**) Batch-corrected PCA of 25 patient-matched hPT/hPC samples and 3 mPT/mPC samples shows location-dependent clustering of human and murine transcriptomes. (**E**) Estimated MCP scores reflecting immune cell infiltration in RNA samples from murine and human PC. ***P < 0.001, Student’s *t* test.

**Figure 8:**
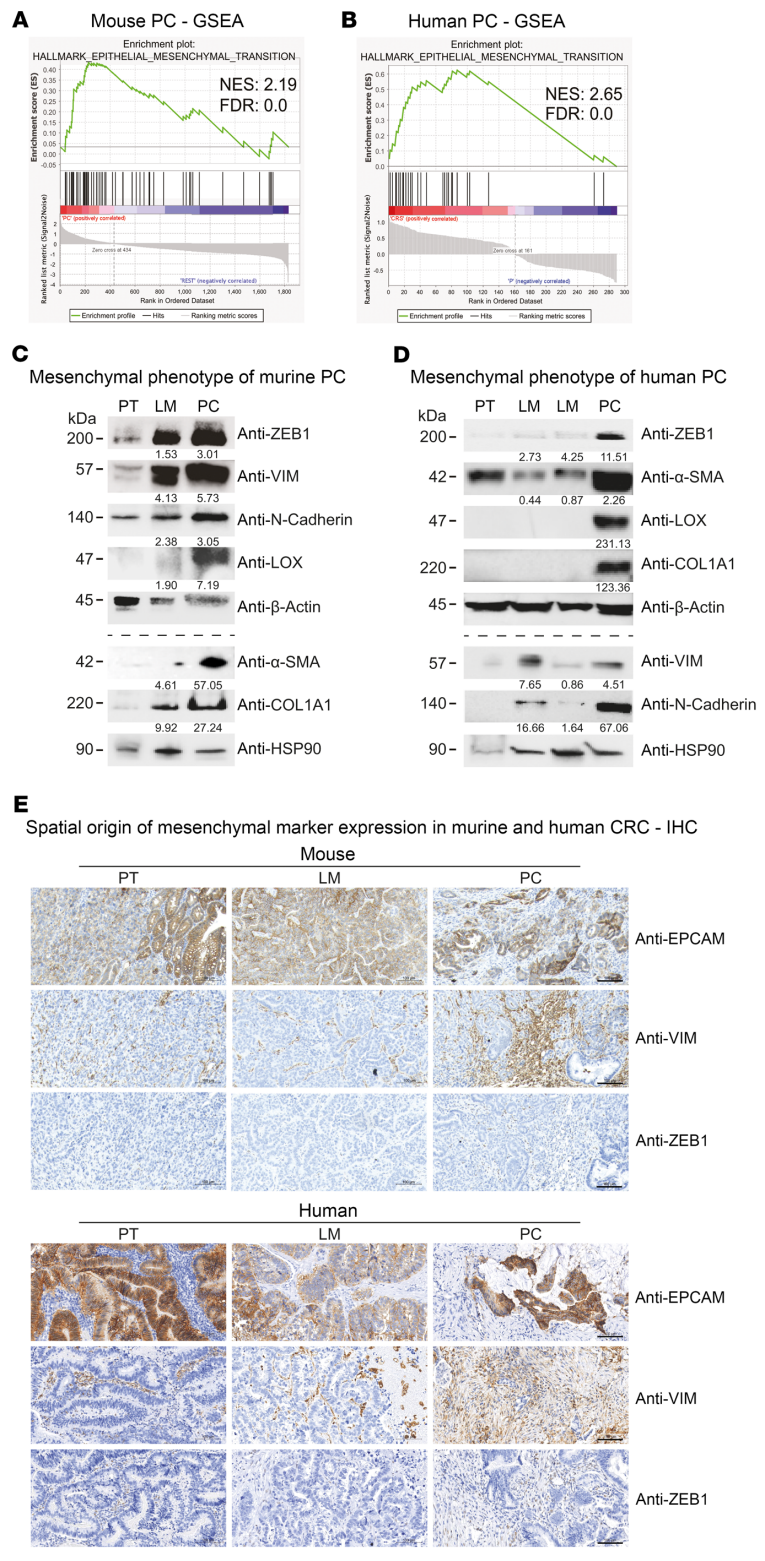
PC is associated with a mesenchymal phenotype in human and murine CRC. (**A** and **B**) GSEA (Signal2Noize) with Hallmark EMT gene signature in murine and human PC. NES, normalized expression score. (**C**) Representative Western blot showing expression of depicted proteins in lysates from murine PT, LM, and PC. Fold change normalized to respective loading control (upper panel, β-actin; lower panel, HSP-90) and PT. The experiment was performed twice. (**D**) Western blot showing expression of depicted proteins in representative lysates from human PT, LM, and PC. Fold change normalized to respective loading control (upper panel, β-actin; lower panel, HSP-90) and PT. The experiment was performed twice. (**E**) Representative IHC of murine and human PT, LM, and PC for EPCAM, VIM, and ZEB1. IHC was performed on 10 tissue samples of each location. Scale bars: 100 μm.

We next tested whether murine multivisceral APTKA CRC mimics human Union for International Cancer Control (UICC) IV CRC. In order to analyze transcriptomic similarities, we used published mRNA-Seq data from human patient-matched PTs (hPT) and PC (hPC) (45) as well as hPT and hLM (NCBI’s Gene Expression Omnibus [GEO] GSE50760) and integrated these data with murine mRNA-Seq data we obtained from 3 PTs (mPT), 4 mLMs, and 3 PC (mPC) samples (Figure 7A). Principal component analysis (PCA) of the mouse data showed localization-dependent clustering, acknowledging the intersite-specific differences in the scRNA-Seq data (Supplemental Figure 7A). To eliminate species-specific differences, we performed batch-corrected PCA and found tumor site–specific clustering of mPT and hPT as well as mLM and hLM (Figure 7B). Next, we compared the immunological TME of murine and human LM by applying the Microenvironment Cell Populations-counter (MCP-counter) deconvolution algorithm (46). Here, we found equivalent proportions of macrophages, B cells, NK cells, and T cells and similar cytotoxicity scores in murine and human LM (Figure 7C). Importantly, strong accordance between human and murine PT and PC was also obtained for the PCA and MCP-counter analysis (Figure 7, D and E). Differential expression analyses revealed 443 overlapping differentially regulated genes (DRGs) between PT and PC from human and murine backgrounds (Supplemental Figure 7B). GSEA in murine tissues indicated upregulation of genes involved in epithelial-mesenchymal transition (EMT) in PC (Figure 8A). Consistently, we also found enrichment of genes involved in EMT in hPC (CRS) (Figure 8B) and could show upregulation of *Snai2*/*Slug* downstream target genes in human and murine PC (Supplemental Figure 8A). Accordingly, we found relevant increases in protein expression of EMT proteins, such as ZEB1, N-cadherin, α-SMA, vimentin, LOX, and COL1A1, in murine and human PC (Figure 8, C and D). To further understand the cellular origin of this increased EMT marker expression, we conducted IHC for EPCAM to stain tumor epithelium as well as VIM and ZEB1 in murine and human samples. In accordance with our results from the scRNA-Seq analysis, we found a strong dominance of the stromal compartment, visualized by a reduction of EPCAM^+^ tumor cells in PC (Figure 8E). Furthermore, VIM as well as ZEB1 expression was not found in the epithelium, but the stromal compartment (Figure 8E). Quantification of VIM^+^ and ZEB1^+^ cells per HPF revealed a marked increase for both markers in murine and human PC (Supplemental Figure 8B). Consequently, the mesenchymal phenotype of PC CRC underlies the increased stromal infiltration. Additionally, the fibrotic phenotype of specifically PC in mouse and human CRC was corroborated by COL1A1 IHC and Masson-Goldner stainings (Supplemental Figure 8C). To gain further insights into whether our murine multivisceral APTKA CRC model also recapitulated human stage IV CRC in terms of cellular composition and TME, we performed scRNA-Seq from human biopsies of 3 primary CRC, 4 LM, and 4 PC tissues. This analysis revealed broadly similar clusters and location-dependent compositions as the murine model (Supplemental Figure 8D). Consistent with proportions obtained from the murine model, PC samples were largely depleted of B cells (Supplemental Figure 8E). We next wanted to ascertain that mouse and human cell types are fully comparable in the TME. When integrating both human and mouse PT/CRC, LM, and PC following conversion of mouse genes to their human orthologues, all main cell types and locations clustered together independently of the species of origin (Supplemental Figure 8F). Accordingly, cell proportions of the combined data further validated the loss of B cells in human and mouse PC, eliminating any potential clustering artifacts (Supplemental Figure 8G). Thus, our results delineate a high similarity of murine LM and PC with human LM and PC and a stroma-dominant mesenchymal phenotype of murine and human PC, emphasizing the high translational potential of the APTKA multivisceral CRC mouse model for further molecular site–specific deconvolution of CRC metastasis and future preclinical evaluation of therapeutic approaches.

### Intraperitoneal application of checkpoint inhibitors reconstitutes effector T cell function in PC and emphasizes the importance of developing site-specific CRC therapy.

**Figure 9:**
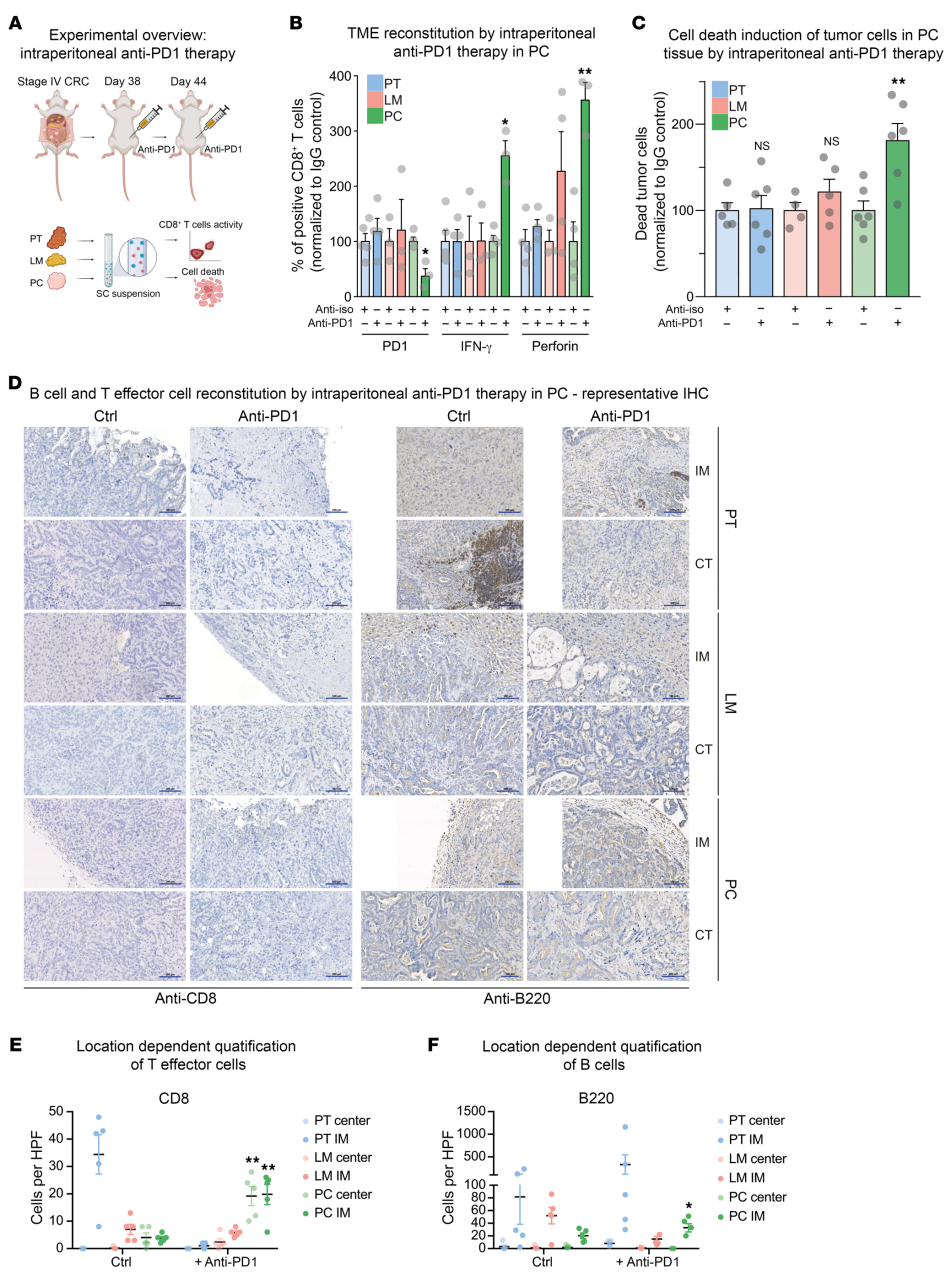
Intraperitoneal application of ICB reconstitutes effector T cell function in PC and emphasizes the importance of a site-specific CRC therapy. (**A**) Experimental overview of stage IV CRC treatment by intraperitoneal application of anti-PD1 therapy. (**B**) Quantification of changes in CD8^+^ T cell regulation upon ICB by FACS. Normalized to IgG control, in percentages. *n* = 3–5 per group. (**C**) Quantification of changes in epithelial cell death (EPCAM^+^Zombie^+^ cells) upon ICB by FACS. Normalized to IgG control, in percentages. *n* = 4–6 per group. (**D**) Representative IHC of murine PT, LM, and PC at the IM and CT for CD8 and b220 with and without anti-PD1 treatment. IHC was performed on 5 tissue samples for each location and antibody. (**E**) Quantification of CD8^+^ cells with and without ICB in PT, LM, and PC tissue at both IM and CT (*n* = 3–5). (**F**) Quantification of b220^+^ cells with and without ICB in PT, LM, and PC tissue at both IM and CT (*n* = 3–5). *P < 0.05; **P < 0.01, Student’s *t* test. Scale bars: 100 μm.

So far, immune checkpoint blockade (ICB) in LM is only shown to be effective in microsatellite instability–high (MSI-high) CRC (47, 48). Based on our results, anti-PD1 therapy should specifically be evaluated in peritoneal CRC metastasis of non-MSI CRC. To demonstrate the translational potential of the APTKA orthotopic mouse model and to verify our presented data, we tested the site-specific reactivation of CD8^+^ T cells by administration of anti-PD1 therapy. Stage IV CRC was established as described before (Figure 1, A and B, and Supplemental Figure 1, A–C). On days 37 and 44, we intraperitoneally treated mice with 300 μg anti-PD1 antibody. Control animals were treated by equivalent IgG isotype control (Figure 9A). Animals were sacrificed on day 56 and PT, LM, and PC tissue was harvested. Function of infiltrative CD8^+^ effector T cells was analyzed using FACS. Importantly, we found substantial reduction of PD1 expression and increased degranulation markers IFN-γ and PRF1 upon ICB specifically in PC tissue, which indicated compartment- and location-dependent therapy response, since CD8^+^ T cells in PT and LM were not substantially activated by ICB (Figure 9B). Furthermore, anti-PD1 application tended to result in increased numbers of infiltrative CD8^+^ T cells and in upregulation of granzyme B and CD107α in PC without statistical significance (Supplemental Figure 9A). In accordance with these results, macroscopic PC load could markedly be reduced by ICB (Supplemental Figure 9B). Specific cell death induction of the epithelial compartment of respective tumor sites was then evaluated using FACS. Importantly, we found marked upregulation of cell death in the tumor cell compartment of PC upon anti-PD1 administration, whereas tumor cells of PT and LM were not affected (Figure 9C). To further investigate the spatial reconstitution of the immunological TME by ICB, we performed IHC for B and cytotoxic T cell infiltration (b220 and CD8). Intriguingly, our findings revealed an enhanced infiltration of CD8^+^ T cells specifically within the PC lesions following anti-PD1 treatment in the tumor center (CT) as well as the invasive margin (IM) while this alteration was not observed in PT and LM (Figure 9, D and E). Quantification of b220 revealed an increase of B cell infiltration especially at the IMs of PC mass, suggesting a potential restoration of B cell–dependent antigen presentation (Figure 9, D and F). These data are in accordance with our scRNA-Seq results, where we have suggested CD8^+^ T cells in PC as location-specific targets for immunomodulation. Hence, our results depict the importance of a further location-specific understanding of CRC metastasis for the development of individualized and location-adapted therapeutic approaches.

## Discussion

To date, single-cell transcriptome studies in CRC have either focused on specific tumor cell populations or on the comparison of 2 tumor sites (6, 49). In this study, we comprehensively mapped the location-dependent cellular landscape of multilocular CRC in an orthotopic, organoid-driven mouse model, which mimics human UICC IV disease with peritoneal and liver metastases. This approach enabled us to decipher intercellular communication networks and metabolic changes during metastasis to different locations. We were able to not only characterize lineage-dependent transformations in tumor cells, but also in low-abundant TME populations such as stromal and immune cells without the influence of therapeutic interventions.

Specifically, we demonstrated the dominance of CSCs in PC, preserving a self-promoting niche by stem cell–supportive intercellular L-R signaling and increased fatty acid metabolism. This was consistent with previous studies that have demonstrated a crucial role of fatty acid metabolism for stemness, maintenance, self-renewal, and therapy resistance of CSCs (20, 50–52). Additionally, this niche is supported by CAFs, potentially leading to increased Wnt and Hippo signaling in respective CSCs. In our model, CAFs and endothelial cells also seem to drive the inflammatory and immune-suppressive phenotype of PC, accompanied by dysfunction of the immunological TME. Our insights into the stromal compartment correspond with published studies, where multiple subtypes of CAFs have been identified in different cancer entities (32, 33, 53–57).

In the metastatic TME, adaptive antitumoral immune cells such as cytotoxic CD8^+^ T cells lose their cytolytic activity and become exhausted, accompanied by upregulation of OXPHOS metabolism. Simultaneously, further cytotoxic cells such as NK cells gain activity in LM and PC. Metabolic reprogramming toward OXPHOS and simultaneous activation of NK cells has already been studied in various diseases (58–60).

Furthermore, we distinguished intermetastatic Th1 CD4^+^ T cell function, which in PC is predominantly governed by immunosuppressive PD-L1 signaling toward CD8^+^ cells and accompanied by a loss of B cell–dependent MHC-II signaling, potentially resembling a disturbed antigen-presenting function of MHC-II^+^ B cells in PC. In a translational approach, we demonstrated strong location-dependent concordance of the orthotopic mouse model with human multilocular CRC metastasis. Our bulk proteomic and transcriptomic analyses suggest congruent deconvolution of the configuration of murine and human LM (61) and PC (45) from both species. Concomitantly, murine and human PC are characterized by increased EMT marker expression originating from a strong stromal infiltration at respective sites. Hence, our data depict explicit site-specific adaptations to local environments during disease progression in the reported mouse model mimicking human multivisceral CRC.

Most of the published stage IV CRC mouse models rely on direct intraperitoneal or intrasplenic injections of permanent tumor cell lines into immunocompetent or immunodeficient mice that do not mimic the physiological route of metastasis (62). Furthermore, permanent cell lines are selected epithelial subpopulations of the original tumor tissue. Therefore, they do not resemble the mutational burden and architecture of the original tumor. In contrast, tumor organoids recapitulate the molecular and architectural features of the original tumor both of mice and humans (63, 64). In our model, CRC cells from invasive PT tissue spread hematogenously and per continuitatem and thereby lead to multivisceral metastatic disease.

In our study, adapted from Fumagalli et al. (10, 11), APTKA organoids were surgically implanted into the subserosa of the cecum of immunocompetent mice. Infiltrative direction is inverted when being compared with the physiological condition of CRC tumorigenesis. Consequently, the process of peritoneal metastasis represents a relatively smaller barrier to traverse when compared with human CRC, where tumor cells must navigate across the basal membrane and the muscular and serosal layers to access the peritoneal space, which could be one explanation for the high percentage of PC obtained in our model. Importantly, we can depict a transluminal epithelial barrier breach already at day 7 after implantation of the organoids. Consequently, tumor cells — for most of the time of the experiment —were already also reinfiltrating from the luminal side toward the colon wall and therefore were exposed to intestinal stimuli similar to that of human CRC. The mutational spectrum of the APTKA organoids used in this project can also be found in human CRC, but accumulation of the whole mutational spectrum is not observed in the majority of CRC patients (CMS4, approximately one-fourth of CRC patients). Still, we could show a strong overlap of murine and human tumor tissue in 2 large patient cohorts of bulk RNA-Seq analyses, regardless of the mutational burden of the human CRC samples. In our stage IV CRC model, orthotopic APTKA implantation was performed into the right-sided colon. Here, we make use of the exclusion of the cecal blind loop from the lower gastrointestinal passage. This facilitates longer experimental time frames until successful metastatic disease occurs without intestinal obstruction by the PT.

Importantly, based on the collected data in this project, we also propose a PC-specific therapeutic approach. In a proof-of-concept experiment, we successfully treated murine APTKA PC CRC by intraperitoneal application of anti-PD1 biologicals. This led to reactivation of exhausted CD8^+^ T cells and increased tumor cell death in intraperitoneal tumors. Macroscopic quantification of tumor mass revealed a successful reduction of intraperitoneal tumor load upon ICB.

Our observations reinforce the necessity for a highly location-specific evaluation of therapeutic interventions, since, e.g., intraperitoneal and systemic application of chemotherapy shows very limited efficacy in PC. To our knowledge, immunomodulatory drugs, e.g., systemic application of ICB, have only been investigated for the treatment of CRLM (65, 66). In this setting, ICB was only successful in MSI-high tumors and therefore is reserved for a small percentage of patients. Further preclinical investigations are needed to elucidate whether locoregional intraperitoneal ICB in combination with, e.g., established treatment protocols or novel immunotherapeutics is a promising therapeutic approach in PC patients.

In summary, we present the comprehensive cellular and molecular landscape of multivisceral CRC and illustrate specific location-dependent reconfiguration of tumor cells and TME. Our findings provide insights into the complex and multifaceted process of metastatic adaptations to local environments and identify exhausted effector CD8^+^ T cells as potential PC-specific therapeutic targets.

## Methods

### Human tumor tissue.

Tumor tissues of patients with primary CRC and metastatic LM and PC were obtained from surgical tumor resections after routine pathological analysis and were either embedded in paraffin, processed for Western blotting, or enzymatically digested for FACS or scRNA-Seq with the Human Tumour Dissociation Kit (Miltenyi Biotec) according to the manufacturer’s instructions.

### Organoids and organoid culture.

APTKA organoids are tumor organoids that are devoid of *Apc*, *Tp53*, and *Tgfbr2* and express constitutively active *Kras* (*KrasG12D*) and an activated/myristoylated isoform of AKT1 (17). APTKA tumor organoids were maintained in basal medium (advanced DMEM-F12 supplemented with penicillin/streptomycin, 10 mmol/L HEPES [Invitrogen], 1× Glutamax [Invitrogen], 1× N2 [Gibco], 1× B27 [Gibco], and 1 mmol/L *N*-acetylcysteine [MilliporeSigma]) supplemented with hygromycin (200 μg/mL) and puromycin (2 μg/mL). The organoids were subcultured in Cultrex basement membrane extract (bio-techne) every 5 to 7 days.

### Mice.

Eight- to 12-week-old male and female C57BL/6J mice from Charles River or CEMT were used as acceptor mice for tumor organoids in the mouse model. Interventions were performed during light cycle. Animals were not specifically fasted.

### Surgical transplantation of organoids under the subserosa of the cecum.

Cultured APTKA organoids were dissociated into a single-cell suspension by mechanical disruption followed by enzymatic digestion for 20 minutes at 37°C using TrypLE (Gibco) and were washed once in ice-cold PBS. Single cells were then resuspended in an ice-cold type I collagen/5× collagen neutralization buffer (4:1 [v/v] ratio) with a concentration of 200,000 cells/10 μL. The 5× collagen neutralization buffer is composed of 2.5 g α MEM powder (5×) and 2 % (wt/vol) NAHCO_3_ in 45 ml Aqua dest and 5 ml of 1M HEPES and set to pH 7.5; 10 μL domes of collagen were plated in 6-well plates. The domes were polymerized for 45 minutes at 37°C. Afterwards, basal medium (Advanced DMEM-F12 supplemented with penicillin/streptomycin, 10 mmol/L HEPES [Invitrogen], 1× Glutamax [Invitrogen], 1× N2 [Gibco], 1× B27 [Gibco], and 1 mmol/L *N*-acetylcysteine [MilliporeSigma]) was supplemented with 10 μM Y-27632 (Tocris Bioscience). The collagen domes were cultured overnight until transplantation. At the day of transplantation, the mice were shaved and anesthetized with 2% (v/v) isoflurane. Analgesia was guaranteed by intraperitoneal injections of 100 μL 0.1 mg/kg buprenorphine. Mice were placed on their backs on a heating pad, and legs were fixed. Isoflurane was lowered to 1.8 % (v/v). A 10 to 15 mm incision was made along the linea alba to open the abdomen. The cecum was placed on wet sterile gauze. A 3 to 4 mm incision through the cecal serosa was made at the end of the cecum in an area without vessels. The serosa was separated from the submucosal layer and a deep pocket of about 1 cm length was created. The pocket was enlarged to a size so that the collagen dome could be deeply embedded in it. The collagen dome was placed under the serosa into the pocket, and the serosa was tightly closed above the collagen dome, securing the collagen dome to be tightly embedded in its pocket. The incision in the serosa was covered with a 20 × 5 mm piece of Seprafilm (Baxter). The cecum was carefully placed back in the abdomen. The peritoneum and abdominal wall were separately closed by a continuous suture. Before awakening, mice were subcutaneously injected with 100 μL metamizole (200 mg/kg) for ongoing analgesia. Drinking water was supplemented with metamizole (5 mg/mL) in 5 % sucrose for the following 3 days.

### Intraperitoneal application of anti-PD1 antibody.

For intraperitoneal injections of anti-PD1 antibody, InVivoMAb anti-mouse PD1 (CD279) (BE0146, BioXCell) was diluted to 3 μg/μL in sterile PBS and 300 μg was intraperitoneally injected on day 37 and day 44 after locoregional APTKA implantation. IgG2a isotype control (BE0089, BioXCell) in the same dilution served as control.

### Laparoscopy.

For tumor staging of PC, laparoscopy was performed. The mice were anesthetized as described before. The abdomen was opened by a small midline incision of 5 mm. For fixation of the endoscope, the incision was surrounded by a pursestring suture. The endoscope was introduced and fixed by the suture. Oxygen was introduced into the abdomen by the endoscope, and tumorigenesis as well as metastasis was monitored.

### PCI.

To stage the PC, we calculated a PCI similar to the human PCI index during laparoscopy. The index is calculated by measuring the amount and the size of PC in the peritoneum. We divided the peritoneum into the upper peritoneum and lower peritoneum and also divided the left and right side and counted the amount of peritoneal metastases on the abdominal wall and the interenteric metastasis. The sizes were divided into small (score 1), middle (score 2), and large metastasis (score 3) or no metastasis (score 0). A small tumor was a tumor with a diameter of 0 to 2 mm, a middle-sized tumor had a diameter of 2 to 4 mm, and a large tumor had a diameter of more than 4 mm. The numbers of the tumors of each part of the 4 quadrants were counted, and the score of each quadrant was calculated as follows: score of quadrant = number of small tumors × 1 + number of middle tumors × 2 + number of large tumors × 3. The PCI was calculated as follows: score of left side of the upper peritoneum + score of the right side of the upper peritoneum + score of the left side of the lower peritoneum + score of the right side of the lower peritoneum.

### H&E, PAS, and Masson-Goldner staining.

Murine and human PT and CRC metastatic tissue was harvested, fixed in 4 % paraformaldehyde for 24 hours, and embedded in paraffin. Paraffin blocks were sliced into 3 μm thick sections. For H&E, PAS, and Masson-Goldner staining, slides were deparaffinized with Rotihistol (Carl Roth) and rehydrated in a descending alcohol series. For H&E staining, slides were counterstained with hematoxylin (Merck) with fixation in warm tap water for 10 minutes. After hematoxylin staining, slides were stained with eosin (Sigma Aldrich). Excess eosin was washed off with 70 % ethanol. For PAS staining, the slides were stained with the Periodic Acid Schiff Stain Kit (Mucin Stain) (Abcam) according to the manufacturer’s instructions. For Masson-Goldner staining, the slides were stained with the Masson-Goldner Staining Kit (Merck) according to the manufacturer’s instructions. For all stainings, slides were dehydrated and mounted with Rotihistokit (Roth). Slides were digitalized with Zeiss Axio Scan Z.1 Microscope Slide Scanner (Zeiss).

### Immunohistology.

For immunohistochemical staining, slides were deparaffinized and rehydrated. Antigen retrieval was performed by pressure cooking in 20 mM citrate buffer (pH 6.0) or T-EDTA (pH 9.0) and the samples were incubated with 3% H_2_O_2_ for 30 minutes in order to reduce endogenous peroxidase activity. After blocking in 5% goat serum (Sigma-Aldrich) for 1 hour, samples were incubated at 4°C overnight with primary antibodies: CD45, EPCAM, CD8, b220, PDGFRB, CD90, VIM, ZEB1, and COL1A1 (see Supplemental Table 2). Slides were washed and primary antibodies were detected using HRP-polymer (Zytomed Systems) according to the manufacturer’s instructions. DABplus (Zytomed Systems) was added, and the slides were counterstained with hematoxylin and mounted with Rotihistokit (Roth).

### Western blotting.

Proteins were isolated after dissociation of whole tumor tissue with a rotor-stator homogenizer with ice-cold RIPA buffer supplemented with protease and phosphatase inhibitors (Santa Cruz Biotechnology Inc. and Roche). Equal amounts of protein (25 μg) were resolved in 4× Laemmli buffer (Bio-Rad), electrophoresed in 10 % Mini-PROTEAN TGX Precast Gels (Bio-Rad), and transferred to PVDF membranes (Bio-Rad). The membranes were blocked with either 5% nonfat dry milk or 5% BSA in T-TBS and were incubated with primary antibodies PDHA1, LDHA, FAS, ACC1, ZEB1, VIM, CDHN, ACTA2, LOX, COL1A1, ACTB, and HSP90 (1:1000 in blocking buffer, see Supplemental Table 1) at 4°C overnight. After washing, secondary HRP-conjugated antibodies (1:1,000 in blocking buffer, Cell Signaling Technology) were incubated for 1 hour at room temperature. Protein expression was visualized with Immobilion Western HRP substrate (Merck Millipore).

### Flow cytometry.

PT, LM, and PC tissue was minced in 1 to 2 mm pieces and was enzymatically digested with the Tumor Dissociation Kit, mouse (Miltenyi) on the gentleMACS Octo Dissociator according to the manufacturer’s instructions. The resulting single-cell suspension was filtered through a 100 μm cell strainer, and erythrocytes were lysed with ACK lysis buffer (Gibco). After cell counting, cells were either stimulated for cytokine analysis or we proceeded with antibody staining. For stimulation, cells were stimulated with Cell Activation Cocktail (BioLegend) for 3 hours and additionally 2 hours together with Brefeldin A (BioLegend). For FACS analysis, cells were first stained with live/dead staining with the Zombie NIR Fixable Viability Kit (BioLegend) and Fc receptors were blocked with Fc block (BioLegend). Afterwards, cells were incubated with fluorescent-labeled antibodies (see Supplemental Table 3) for surface staining in FACS buffer (PBS, 4 % FCS, 0.05% sodium azide) for 20 minutes at 4°C. For intracellular staining, cells were fixed in fixation buffer (Foxp3 staining buffer, Invitrogen) for 1 hour and washed in permeabilization buffer 2 times. The cells were stained with intracellular antibodies for 20 minutes at 4°C in permeabilization buffer and washed once again in permeabilization buffer. The analyses were performed on a BD Fortessa flow cytometer.

### Tumor cell isolation.

Tumor cells were isolated from single-cell suspensions of whole tumor tissue with the Tumor Cell Isolation Kit (Miltenyi Biotec) according to the manufacturer’s protocol.

### RNA-Seq.

RNA isolation from tumor tissue was performed with the RNeasy MiniKit (Qiagen) according to the manufacturer’s protocol. RNA samples were sequenced by the standard Illumina protocol to create raw sequence files (.fastq files) at Genewiz. Mm39 genome indexes were created using STAR 2.7.9 using --runMode genomeGenerate --sjdbOverhang 100. Fastq files were aligned to the mm39 genome using STAR 2.7.9a using --outSAMtype BAM --outSAMunmapped Within --outSAMattributes Standard. Quantification was carried out using featureCounts 1.6.2 using -p -B -C -Q 10 --primary -s 0 as parameters. Differential analysis was carried out using DESeq2 using design~=condition and standard subsequent commands. Normalization was performed using variance stabilization transformation (vst). Row *z* scores were computed using the following formula: *z* = *t*(scale(*t*(<batchj corrected vst object>),scale = *T*, center = *T*)). Clustering was performed using the heatmap.2 function. Visual inspection of the data was performed using PCA. GSEA analyses were performed using GSEA 4.1.0. Raw.fastq files can be accessed via GEO GSE202454.

### RNA-Seq data integration.

Published human CRC data were retrieved from the Sequence Read Archive database (SRA PRJNA764756). Only PT data were retained from this data set. Human CRC data were processed as described above except that they were aligned to the hg38 genome. CMS classification was performed using MmCMS 0.1.0 using the MmCMS-A function according to the developer’s protocol (67). Raw counts from human and mouse were merged at the gene level by converting mouse to human gene symbols using biomaRt, retaining matching genes only. This yielded 17,843 genes available for analysis. Merged data were subsequently analyzed using DESeq2 using design~=batch+condition and subsequent standard commands, where batch represented either human or mouse data. Batch correction was performed using limma:removeBatchEffect on experiment assays following vst. Bias against assigned conditions was performed by swapping conditions between human and mouse data, which resulted in converse groupings for PC/CRS and PT/P samples, which were therefore conducive to no bias. For deconvolution and MCP counter analyses, the deconvolute function of the Cibersort R package was used, using quantiseq and mcp_counter on normalized DESeq2 counts. GSEAs were evaluated (https://www.gsea-msigdb.org/gsea/).

### Mouse scRNA-Seq.

For whole tumor samples, samples from PT, LM, and PC were processed for RNA isolation, library preparation, scRNA-Seq, and count data generation by Singleron Biotechnologies via established protocols. Data were obtained as raw count tables as well as.fastq files (see GEO GSE220507). Tissues from 6 mice per location were pooled before single cell dissociation. Raw count data for all 3 samples were obtained as h5Seurat format.

For analyses of infiltrative lymphocytes, single-cell suspensions (see also *Flow cytometry*) from APTKA PT, LM, and PC samples (cells from 6 mice per location) were pooled before tumor cell dissociation with the Tumor Dissociation Kit, mouse (Miltenyi). Single-cell suspensions were subjected to single-cell sorting using a MoFlow Astrios cell sorter. After exclusion of dead and CD45^–^ cells, all live CD45^+^CD11b^–^ cells were sorted and directly processed for 10X (10X Genomics) protocols.

### scRNA-Seq data analysis.

Data collection and analyses were performed in an unsupervised manner, but not blinded to the location of the samples.

scRNA-Seq was conducted using the Chromium Next GEM Single Cell 3′ Kit, version 3.1. Up to 60,000 cells were processed per reaction. The dual index protocol was performed following the manufacturer’s instructions. The samples were sequenced on a NextSeq 1000/2000 Illumina system using P2 reagent with 100 cycles. Libraries were diluted to be loaded at 1,200 pM. Dragen software, version 3.8.4, was used for sample demultiplexing to obtain paired end reads with the unique molecular identifier (UMI) and cell barcode information on read 1 and gene sequences on read 2 of the result.fastq files. After achieving a sequencing depth of around 20,000 reads per cell,.fastq files were analyzed using cellranger-6.1.2 with the Gencode mouse release M24 reference genome for read alignment. Filtered raw-count matrices were further analyzed using the Seurat (version 4.1.0) R package for data integration and unbiased clustering of single cell transcriptomes (68). Low-quality single-cell libraries with less than 250 and more than 4,000 detected genes and more than 20% mitochondrial counts were excluded in whole tumor samples. Here, we analyzed 10,398 cells after quality control. In addition, low-quality clusters with low numbers of detected genes and high percentages of mitochondrial genes and/or nearly exclusive ribosomal gene expression that lacked expression of biologically relevant cell type markers were removed after clustering. Consequently, data analysis was performed with 8,094 annotated high-quality cells. For sorted immune cells, the mitochondrial counts threshold was set to 10%, which led to the analysis and annotation of 15,714 cells after quality control.

Data sets from different localizations were integrated using the IntegrateData function in Seurat. Data were centered and scaled using the ScaleData function. PCA was used for dimensionality reduction and the K-nearest neighbor (KNN) graph was constructed using the first 30 principal components. Cells were clustered using the Louvain algorithm and embedded in 2D space (30 principal components) using UMAP. Differentially expressed genes between clusters were identified using Wilcoxon’s rank sum test, and clusters were assigned to cell types according to expressed marker genes and their similarity to known cell types. We merged transcriptional clusters if their average gene expression profiles were highly correlated and if they were characterized by similar cell type–specific marker genes. Plots were generated with Seurat or custom R script using ggplot2 v3.3.5. L-R interactions were calculated and plotted using CellChat, version 1.1.3, as already described (22). Minimal cell count per cluster for potential cell-cell interactions was set as default. Metabolic scoring was analyzed using scMetabolism, version 0.2.1, R-package, as demonstrated before (6). For adaptation to the murine species, gene symbols in the package’s.gmt files were converted to murine gene symbols using biomaRt, version 2.50.3.

### Statistics.

Data are represented as mean ± SEM. Significance was calculated by 2-tailed Student’s *t* test, hypergeometric test, or Benjamini-Hochberg procedure as indicated where appropriate. Statistical significance was set as *P* < 0.05.Sample sizes are indicated where appropriate.

### Study approval.

The human study was approved by the local ethics committee of the University of Freiburg (21-1162), was conducted according to the Declaration of Helsinki, and was performed in accordance with ARRIVE guidelines. All subjects provided written, informed consent. All experiments were performed in accordance with local guidelines and regulations. The study was approved by the government of Upper Palatine, Bavaria, and Ministerium Freiburg (G19/41, G20/172, G23/088, X18/07K.

### Data and materials availability.

All raw data, code, and materials are available from the authors upon request. Raw data have been deposited in the GEO database (GSE202454 and GSE220507). Values for all data points in graphs are reported in the Supporting Data Values file.

## Author contributions

CB and RK conceived and conducted the project and wrote the manuscript. CB and BM established the PC mouse model. BM and L Marx performed mouse experiments, immunohistology, FACS analysis, and laparoscopy. RS, BM, M Prinz, and JL established the scRNA-Seq pipeline and performed scRNA-Seq. CB and PC performed bioinformatics. JL, OG, RF, and CL helped in establishment of the mouse model. L Miketiuk, NR, LS, and RF performed experiments. SFF, DJ, PAH, HN, AJ, and EAB helped in human studies. FRG and M Pesic established the APTKA organoids.

## Supplementary Material

Supplemental data

Unedited blot and gel images

Supplemental video 1

Supplemental video 2

Supporting data values

## Version 1 12/28/2023

In-Press Preview

## Version 2 03/01/2024

Electronic publication

## Version 3 03/12/2024

Added Lisa Marx to authorship note
