# Supplementary material for: Single-cell deconvolution reveals high lineage- and location-dependent heterogeneity in mesenchymal multivisceral stage 4 colorectal cancer
Source: J Clin Invest. 2023 Dec 28;134(5):e169576. doi: 10.1172/JCI169576 (PMC10904044; doi:10.1172/JCI169576)
Supplement: Supplemental data [file jci-134-169576-s024.pdf]

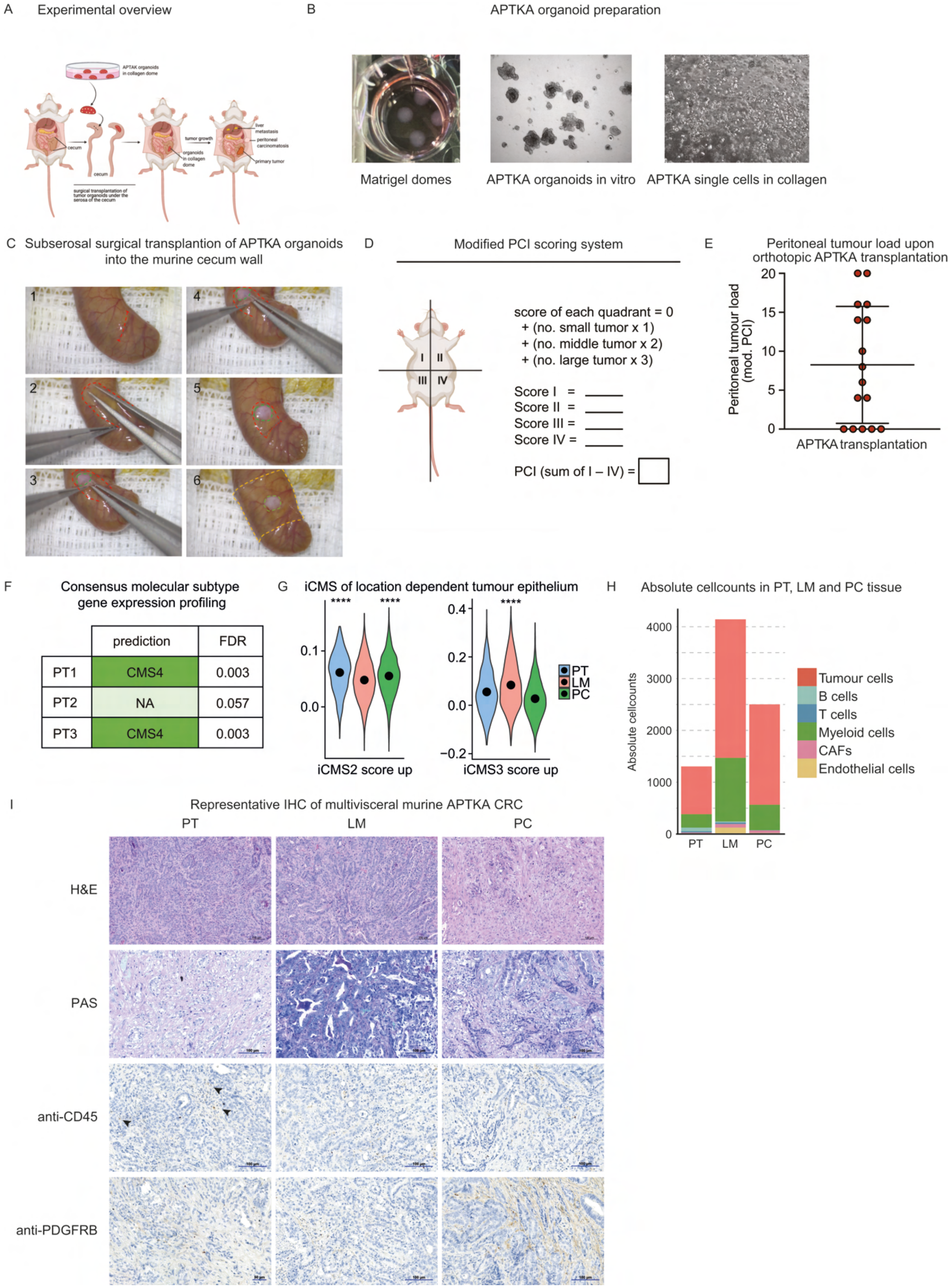
A
Experimental overview
APTAK organoids in collagen dome
cecum
tumor growth
organoids in collagen dome
liver metastasis
peritoneal carcinomatosis
primary tumor
surgical transplantation of tumor organoids under the serosa of the cecum
B
APTKA organoid preparation
Matrigel domes
APTKA organoids in vitro
APTKA single cells in collagen
C
Subserosal surgical transplantion of APTKA organoids into the murine cecum wall
1
2
3
4
5
6
D
Modified PCI scoring system
I
II
III
IV
score of each quadrant = 0
+ (no. small tumor x 1)
+ (no. middle tumor x 2)
+ (no. large tumor x 3)
Score I =
Score II =
Score III =
Score IV =
PCI (sum of I – IV) =
E
Peritoneal tumour load upon orthotopic APTKA transplantation
Peritoneal tumour load (mod. PCI)
0
5
10
15
20
APTKA transplantation
F
Consensus molecular subtype gene expression profiling
prediction
FDR
PT1
CMS4
0.003
PT2
NA
0.057
PT3
CMS4
0.003
G
iCMS of location dependent tumour epithelium
****
****
0.1
0.0
iCMS2 score up
****
0.4
0.2
0.0
−0.2
iCMS3 score up
PT
LM
PC
H
Absolute cellcounts in PT, LM and PC tissue
Absolute cellcounts
0
1000
2000
3000
4000
PT
LM
PC
Tumour cells
B cells
T cells
Myeloid cells
CAFs
Endothelial cells
I
Representative IHC of multivisceral murine APTKA CRC
PT
LM
PC
H&E
PAS
anti-CD45
anti-PDGFRB

Supplementary Figure 1: A distinct cellular and functional landscape of murine primary CRC, liver metastases and peritoneal carcinomatosis

A. Experimental overview for orthotopic subserosal APTKA transplantation. **B.** Representative images of APTKA organoids in matrigel domes during cultivation in vitro (left panel). Day six after passaging (middle panel). Single cells from APTKA organoids embedded in 10µl collagen dome before one day before surgical subserosal cecum transplantation into mice (right panel). **C.** Representative images of the surgical subserosal transplantation of APTKA tumour organoids into the cecum. 1. The cecum is exteriorized and embedded on a wet pad. The red dashes line marks the incision line of the cecum serosa. 2 After incision, the serosa is lifted and a deep subserosal pocket which can fully cover the 10µl collagen dome containing the APTKA organoid cells is formed (red dashed line). 3. Placement of the collagen dome (green dashed line) into the preformed subserosal pocket. 4. Deep implantation of the collagen dome for complete coverage by the cecum serosa. 5. Deeply implanted and fully covered collagen dome in the subserosal pocket. 6. Pre-cut rectangle of an adhesion barrier (yellow dashed line) is placed over the transplantation site and the cecum is wrapped with the film to secure the collagen dome and to prevent adhesions during wound healing. For detailed surgical steps, also see Movie S1. **D.** Schematic representation of the modified peritoneal carcinomatosis index (PCI). **E.** Laparoscopically analysed PCI eight weeks after orthotopic APTKA transplantation. **F.** Consensus molecular subtype of PT according to bulk transcriptome-based analyses. **G.** Violin plot depicting expression of scores for marker genes of iCMS2 and iCMS3. ****$p<0.0001$, Student's T-test. **H.** Absolute numbers for respective celltypes in PT, LM and PC. **I.** H&E staining, periodic acid–Schiff reaction (PAS) and IHC of murine PT, LM and PC for CD45 (immune cells) and PDGFRB (stromal cells). Histology and IHC was performed on 10 slides for each location.

A

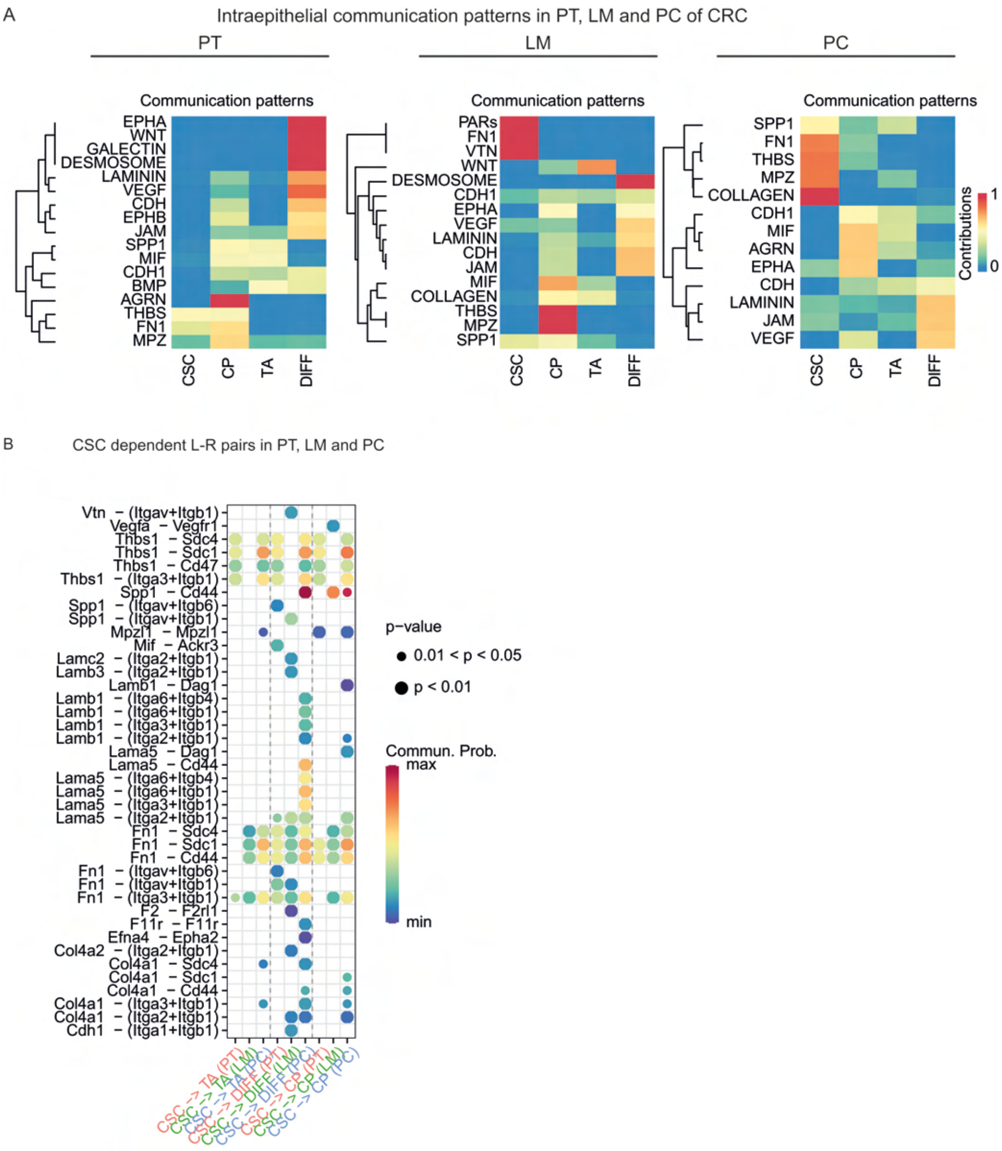

Intraepithelial communication patterns in PT, LM and PC of CRC
PT
LM
PC
Communication patterns
EPHA
WNT
GALECTIN
DESMOSOME
LAMININ
VEGF
CDH
EPHB
JAM
SPP1
MIF
CDH1
BMP
AGRN
THBS
FN1
MPZ
PARs
VTN
COLLAGEN
CSC
CP
TA
DIFF
Contributions
1
0
B
CSC dependent L-R pairs in PT, LM and PC
Vtn - (Itgav+Itgb1)
Vegfa - Vegfr1
Thbs1 - Sdc4
Thbs1 - Sdc1
Thbs1 - Cd47
Thbs1 - (Itga3+Itgb1)
Spp1 - Cd44
Spp1 - (Itgav+Itgb6)
Spp1 - (Itgav+Itgb1)
Mpzl1 - Mpzl1
Mif - Ackr3
Lamc2 - (Itga2+Itgb1)
Lamb3 - (Itga2+Itgb1)
Lamb1 - Dag1
Lamb1 - (Itga6+Itgb4)
Lamb1 - (Itga6+Itgb1)
Lamb1 - (Itga3+Itgb1)
Lamb1 - (Itga2+Itgb1)
Lama5 - Dag1
Lama5 - Cd44
Lama5 - (Itga6+Itgb4)
Lama5 - (Itga6+Itgb1)
Lama5 - (Itga3+Itgb1)
Lama5 - (Itga2+Itgb1)
Fn1 - Sdc4
Fn1 - Sdc1
Fn1 - Cd44
Fn1 - (Itgav+Itgb6)
Fn1 - (Itgav+Itgb1)
Fn1 - (Itga3+Itgb1)
F2 - F2rl1
F11r - F11r
Efna4 - Epha2
Col4a2 - (Itga2+Itgb1)
Col4a1 - Sdc4
Col4a1 - Sdc1
Col4a1 - Cd44
Col4a1 - (Itga3+Itgb1)
Col4a1 - (Itga2+Itgb1)
Cdh1 - (Itga1+Itgb1)
CSC -> TA (PT)
CSC -> TA (LM)
CSC -> TA (PC)
CSC -> DIFF (PT)
CSC -> DIFF (LM)
CSC -> DIFF (PC)
CSC -> CP (PT)
CSC -> CP (LM)
CSC -> CP (PC)
p-value
0.01 < p < 0.05
p < 0.01
Commun. Prob.
max
min

Supplementary Figure 2: Location-specific metabolic reprogramming of the STEM compartment in multivisceral CRC

A. Intraeptihelial communication patterns between cellular subtypes. Heatmap shows the correspondence between the inferred patterns and cell subtypes, as well as signalling pathways. **B.** Location dependent comparison of the significant ligand-receptor pairs between CSC, CP, TA and DIFF. The dot color reflects communication probabilities and dot size represents computed p-values. Empty space means the communication probability is zero.

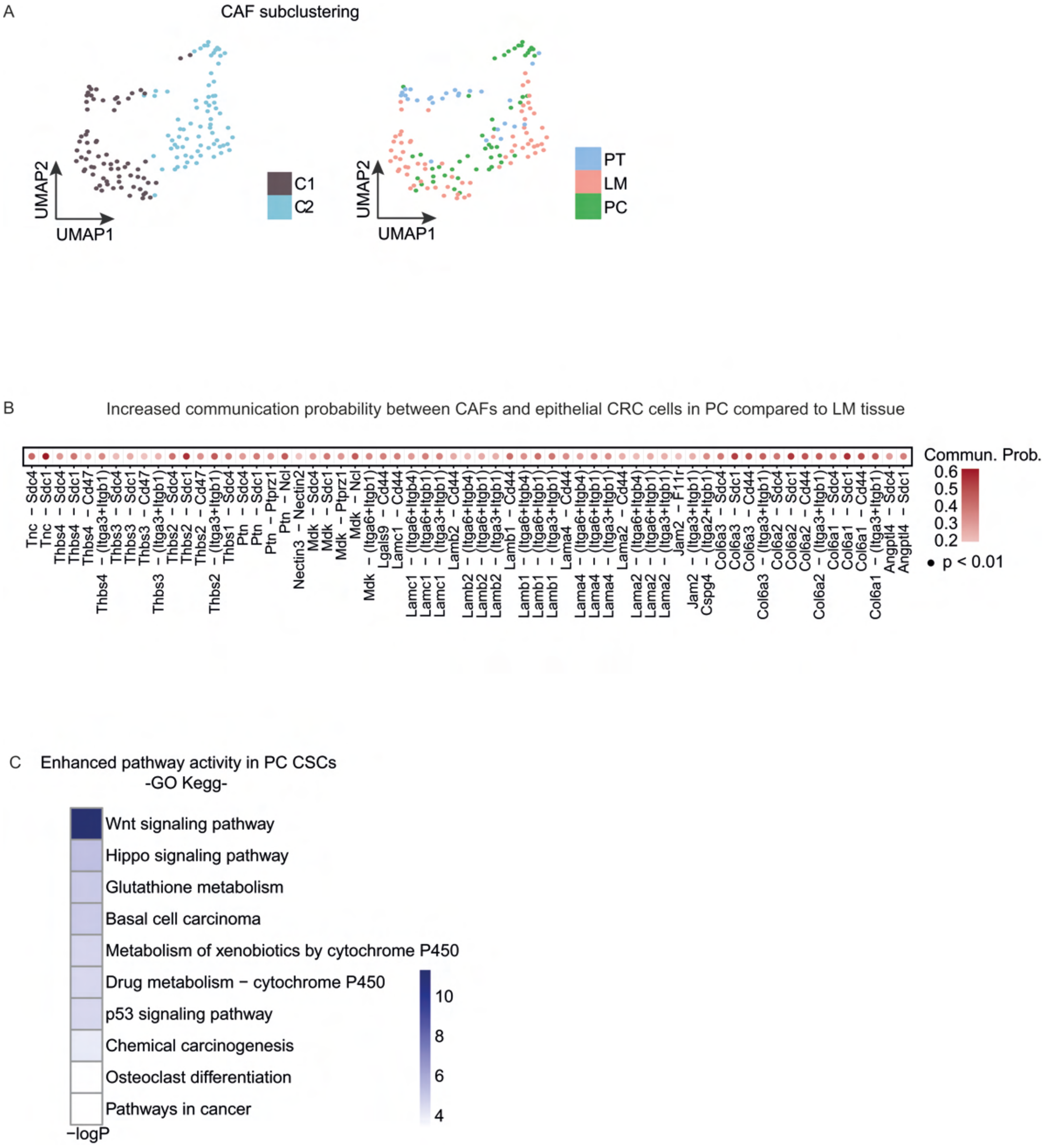
A
CAF subclustering
UMAP2
UMAP1
C1
C2
UMAP2
UMAP1
PT
LM
PC
B
Increased communication probability between CAFs and epithelial CRC cells in PC compared to LM tissue
Tnc − Sdc4
Tnc − Sdc1
Thbs4 − Sdc4
Thbs4 − Sdc1
Thbs4 − Cd47
Thbs4 − (Itga3+Itgb1)
Thbs3 − Sdc4
Thbs3 − Sdc1
Thbs3 − Cd47
Thbs3 − (Itga3+Itgb1)
Thbs2 − Sdc4
Thbs2 − Sdc1
Thbs2 − Cd47
Thbs2 − (Itga3+Itgb1)
Thbs1 − Sdc4
Ptn − Sdc4
Ptn − Sdc1
Ptn − Ptprz1
Ptn − Ncl
Nectin3 − Nectin2
Mdk − Sdc4
Mdk − Sdc1
Mdk − Ptprz1
Mdk − Ncl
Mdk − (Itga6+Itgb1)
Lgals9 − Cd44
Lamc1 − Cd44
Lamc1 − (Itga6+Itgb4)
Lamc1 − (Itga6+Itgb1)
Lamc1 − (Itga3+Itgb1)
Lamb2 − Cd44
Lamb2 − (Itga6+Itgb4)
Lamb2 − (Itga6+Itgb1)
Lamb2 − (Itga3+Itgb1)
Lamb1 − Cd44
Lamb1 − (Itga6+Itgb4)
Lamb1 − (Itga6+Itgb1)
Lamb1 − (Itga3+Itgb1)
Lama4 − Cd44
Lama4 − (Itga6+Itgb4)
Lama4 − (Itga6+Itgb1)
Lama4 − (Itga3+Itgb1)
Lama2 − Cd44
Lama2 − (Itga6+Itgb4)
Lama2 − (Itga6+Itgb1)
Lama2 − (Itga3+Itgb1)
Jam2 − F11r
Jam2 − (Itga3+Itgb1)
Cspg4 − (Itga2+Itgb1)
Col6a3 − Sdc4
Col6a3 − Sdc1
Col6a3 − Cd44
Col6a3 − (Itga3+Itgb1)
Col6a2 − Sdc4
Col6a2 − Sdc1
Col6a2 − Cd44
Col6a2 − (Itga3+Itgb1)
Col6a1 − Sdc4
Col6a1 − Sdc1
Col6a1 − Cd44
Col6a1 − (Itga3+Itgb1)
Angptl4 − Sdc4
Angptl4 − Sdc1
Commun. Prob.
0.6
0.5
0.4
0.3
0.2
p < 0.01
C
Enhanced pathway activity in PC CSCs
-GO Kegg-
Wnt signaling pathway
Hippo signaling pathway
Glutathione metabolism
Basal cell carcinoma
Metabolism of xenobiotics by cytochrome P450
Drug metabolism − cytochrome P450
p53 signaling pathway
Chemical carcinogenesis
Osteoclast differentiation
Pathways in cancer
−logP
10
8
6
4

Supplementary Figure 3: Inter-metastatic differences in stromal cell dynamics during CRC metastasis

A. UMAP plot of CAFs subclusters identified by joint application of RCA and CCA and color-coded by cluster (left panel). UMAP plot of CAFs color-coded by location (right panel). **B.** Significantly increased communication probabilities for L-R pairs between CAFs and tumour cells in LM and PC. The colour represents the communication probability, dot size represents significancy levels. **C.** Unbiased KEGG pathway enrichment analysis with differentially regulated genes (p adj. <0.05) in PC CSCs compared to LM CSCs.

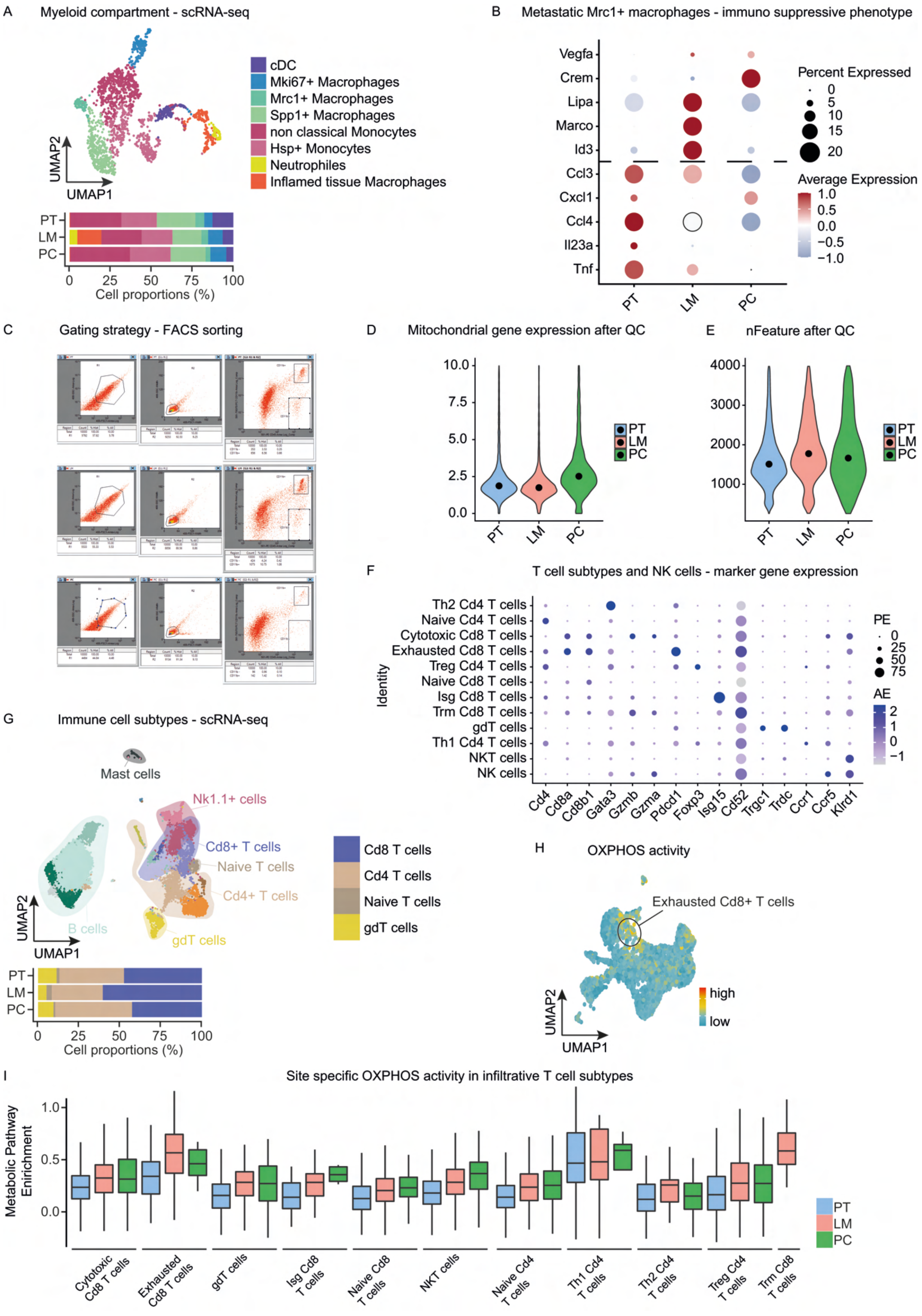

A Myeloid compartment - scRNA-seq
cDC
Mki67+ Macrophages
Mrc1+ Macrophages
Spp1+ Macrophages
non classical Monocytes
Hsp+ Monocytes
Neutrophiles
Inflamed tissue Macrophages
UMAP2
UMAP1
PT
LM
PC
0 25 50 75 100
Cell proportions (%)
B Metastatic Mrc1+ macrophages - immuno suppressive phenotype
Vegfa
Crem
Lipa
Marco
Id3
Ccl3
Cxcl1
Ccl4
Il23a
Tnf
PT LM PC
Percent Expressed
0 5 10 15 20
Average Expression
1.0 0.5 0.0 −0.5 −1.0
C Gating strategy - FACS sorting
D Mitochondrial gene expression after QC
10.0 7.5 5.0 2.5 0.0
PT LM PC
E nFeature after QC
4000 3000 2000 1000
PT LM PC
F T cell subtypes and NK cells - marker gene expression
Th2 Cd4 T cells
Naive Cd4 T cells
Cytotoxic Cd8 T cells
Exhausted Cd8 T cells
Treg Cd4 T cells
Naive Cd8 T cells
Isg Cd8 T cells
Trm Cd8 T cells
gdT cells
Th1 Cd4 T cells
NKT cells
NK cells
Identity
Cd4 Cd8a Cd8b1 Gata3 Gzmb Gzma Pdcd1 Foxp3 Isg15 Cd52 Trgc1 Trdc Ccr1 Ccr5 Klrd1
PE
0 25 50 75
AE
2 1 0 −1
G Immune cell subtypes - scRNA-seq
Mast cells
Nk1.1+ cells
Cd8+ T cells
Naive T cells
Cd4+ T cells
B cells
gdT cells
Cd8 T cells
Cd4 T cells
Naive T cells
gdT cells
UMAP2
UMAP1
PT
LM
PC
0 25 50 75 100
Cell proportions (%)
H OXPHOS activity
Exhausted Cd8+ T cells
high
low
UMAP2
UMAP1
I Site specific OXPHOS activity in infiltrative T cell subtypes
Metabolic Pathway Enirichment
1.0 0.5 0.0
Cytotoxic Cd8 T cells
Exhausted Cd8 T cells
gdT cells
Isg Cd8 T cells
Naive Cd8 T cells
NKT cells
Naive Cd4 T cells
Th1 Cd4 T cells
Th2 Cd4 T cells
Treg Cd4 T cells
Trm Cd8 T cells
PT
LM
PC

Supplementary Figure 4: Location-specific anti-tumoural immunity during CRC metastasis

A. UMAP plot of myeloid cell subtypes identified by joint application of RCA and CCA and color-coded by cell type (upper panel). Proportions of myeloid cell subtypes in PT, LM and PC (lower panel) on average are shown. **B.** Expression of genes involved in polarization of *Mrc1*$^{+}$ macrophages from PT, LM and PC, centred to the average expression of each gene across all locations. The dot size represents the proportion of expressing cells in each cluster. **C.** Gating strategy for FACS guides isolation of CD45^{+} / CD11b^{-} immune cells from whole tumour single cell suspensions from PT (upper panel), LM (middle panel) and PC (lower panel). **D.** Mitochondrial gene expression after QC for each sample depicted as violin plot. **E.** nFeature after QC for each sample depicted as violin plot. **F.** Canonical marker gene expression for multiple T cell subtypes and NK cells centred to the average expression of each gene across cells. The dot size represents the proportion of expressing cells in each cluster. PE: percent expressed. AE: average expression. **G.** UMAP plot of B cells, mast cells, T cells and NK cells identified by joint application of RCA and CCA and color-coded by cell type (upper panel). Proportions of T cell subtypes in PT, LM and PC tissue (lower panel) on average are shown. **H.** UMAP of metabolic scores for OXPHOS in T cell subtypes. **I.** Metabolic activity analysis in the T cell compartment of PT, LM and PC on cell subtype level. Metabolic score depicted as boxplot.

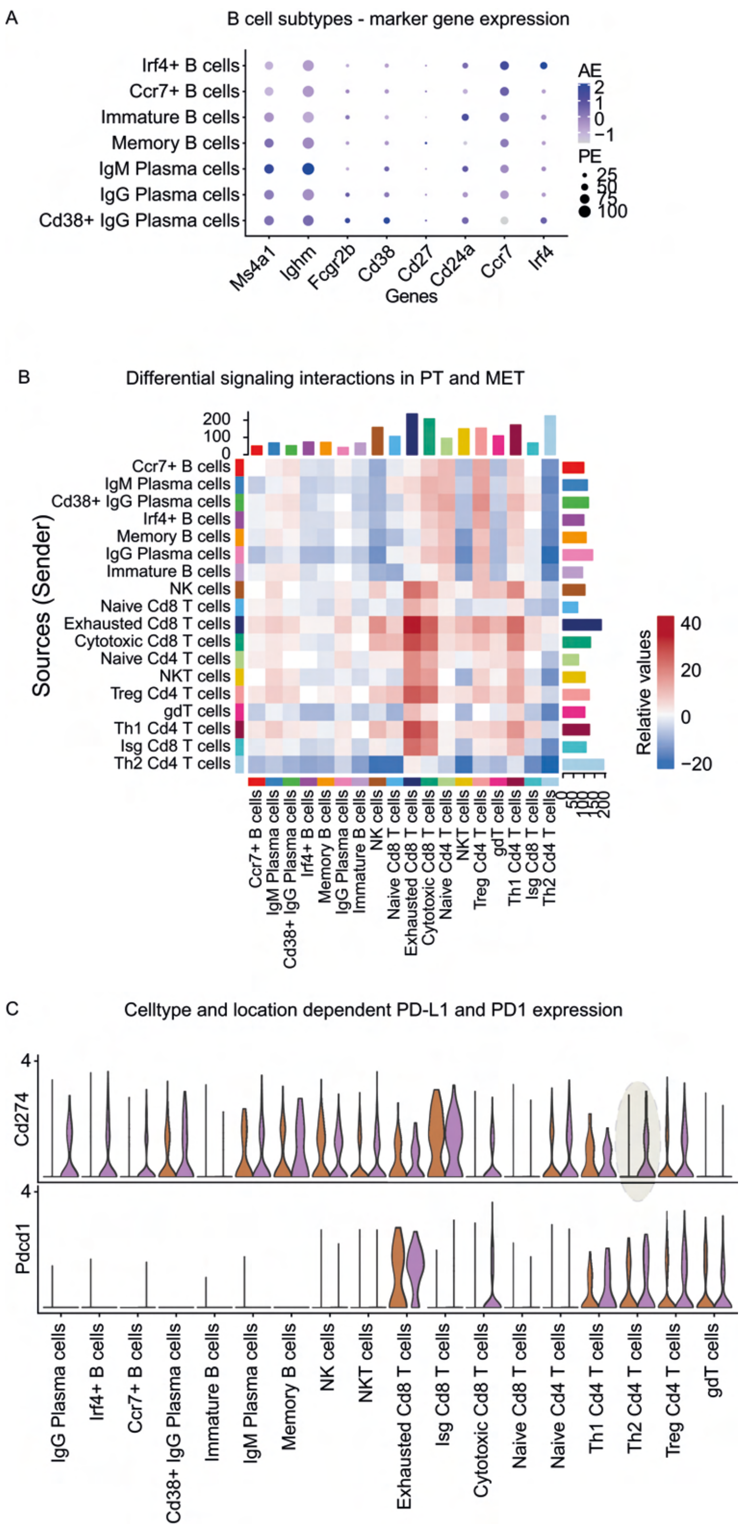
A
B cell subtypes - marker gene expression
Irf4+ B cells
Ccr7+ B cells
Immature B cells
Memory B cells
IgM Plasma cells
IgG Plasma cells
Cd38+ IgG Plasma cells
Ms4a1
Ighm
Fcgr2b
Cd38
Cd27
Cd24a
Ccr7
Irf4
Genes
AE
PE
B
Differential signaling interactions in PT and MET
Sources (Sender)
NK cells
Naive Cd8 T cells
Exhausted Cd8 T cells
Cytotoxic Cd8 T cells
Naive Cd4 T cells
NKT cells
Treg Cd4 T cells
gdT cells
Th1 Cd4 T cells
Isg Cd8 T cells
Th2 Cd4 T cells
Relative values
C
Celltype and location dependent PD-L1 and PD1 expression
Cd274
Pdcd1

Supplementary Figure 5: Impairment of B cell networks in metastasized CRC

A. Canonical marker gene expression for multiple B cell subtypes centred to the average expression of each gene across all cells. The dot size represents the proportion of expressing cells in each cluster. PE: percent expressed. AE: average expression. **B.** Heatmap depicting the differential number of interactions between B cell and T cell subtypes in PT and MET. Colours represent relative values. **C.** Expression level of *Cd274* and *Pdcd1* in B cell and T cell subtypes from PT and MET depicted as violin plot.

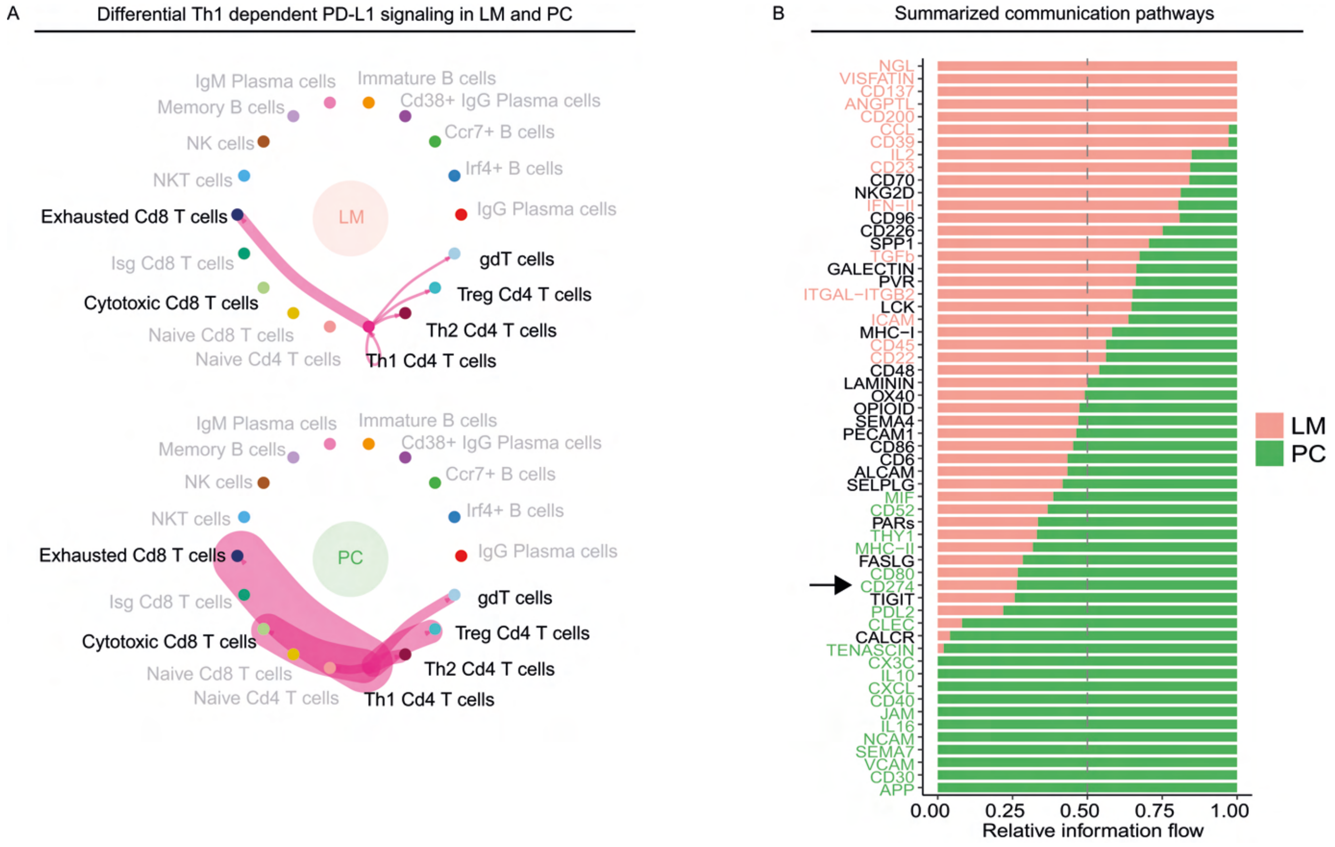

A
Differential Th1 dependent PD-L1 signaling in LM and PC
IgM Plasma cells
Immature B cells
Memory B cells
Cd38+ IgG Plasma cells
NK cells
Ccr7+ B cells
NKT cells
Irf4+ B cells
Exhausted Cd8 T cells
LM
IgG Plasma cells
Isg Cd8 T cells
gdT cells
Cytotoxic Cd8 T cells
Treg Cd4 T cells
Naive Cd8 T cells
Th2 Cd4 T cells
Naive Cd4 T cells
Th1 Cd4 T cells
PC
B
Summarized communication pathways
NGL
VISFATIN
CD137
ANGPTL
CD200
CCL
CD39
IL2
CD23
CD70
NKG2D
IFN−II
CD96
CD226
SPP1
TGFb
GALECTIN
PVR
ITGAL−ITGB2
LCK
ICAM
MHC−I
CD45
CD22
CD48
LAMININ
OX40
OPIOID
SEMA4
PECAM1
CD86
CD6
ALCAM
SELPLG
MIF
CD52
PARs
THY1
MHC−II
FASLG
CD80
CD274
TIGIT
PDL2
CLEC
CALCR
TENASCIN
CX3C
IL10
CXCL
CD40
JAM
IL16
NCAM
SEMA7
VCAM
CD30
APP
0.00
0.25
0.50
0.75
1.00
Relative information flow
LM
PC

Supplementary Figure 6: Inter-metastatic alterations of adaptive immune responses multivisceral CRC

A. Unbiased overall information flow of signalling networks by summarizing all the communication probabilities in respective network. All the significant signalling pathways were ranked based on their differences of overall information flow within the inferred networks between LM and PC. Signalling pathways coloured by red are more enriched in LM, pathways coloured by green were enriched in PC. **B.** Significant ligand-receptor pairs of the PD-L1 signalling network between Th1 $CD4^+$ T cells and other T cell subtypes in LM and PC. The edge width represents MHC-II dependent communication probability.

A

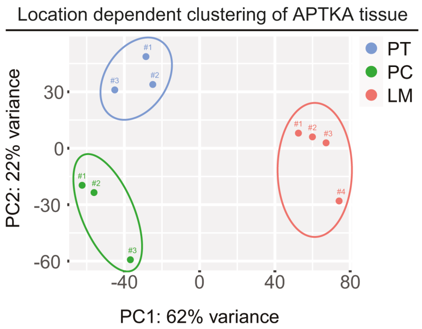
Location dependent clustering of APTKA tissue
PT
PC
LM
PC2: 22% variance
PC1: 62% variance
30
0
-30
-60
-40
0
40
80
#1
#2
#3
#4

B

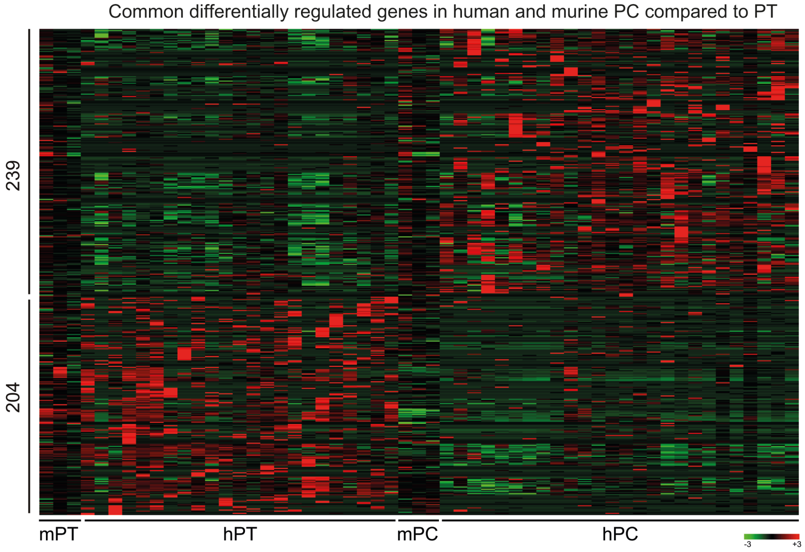
Common differentially regulated genes in human and murine PC compared to PT
239
204
mPT
hPT
mPC
hPC
-3
+3

Supplementary Figure 7: Murine multivisceral APTKA CRC mimics human stage IV CRC

A. Principal component analysis (PCA) of three PT, three LM and four PC tissues show location dependent clustering of transcriptomes. **B.** Heatmap depicting common differentially regulated genes in human and murine PC compared to PT. p adj. <0.05.

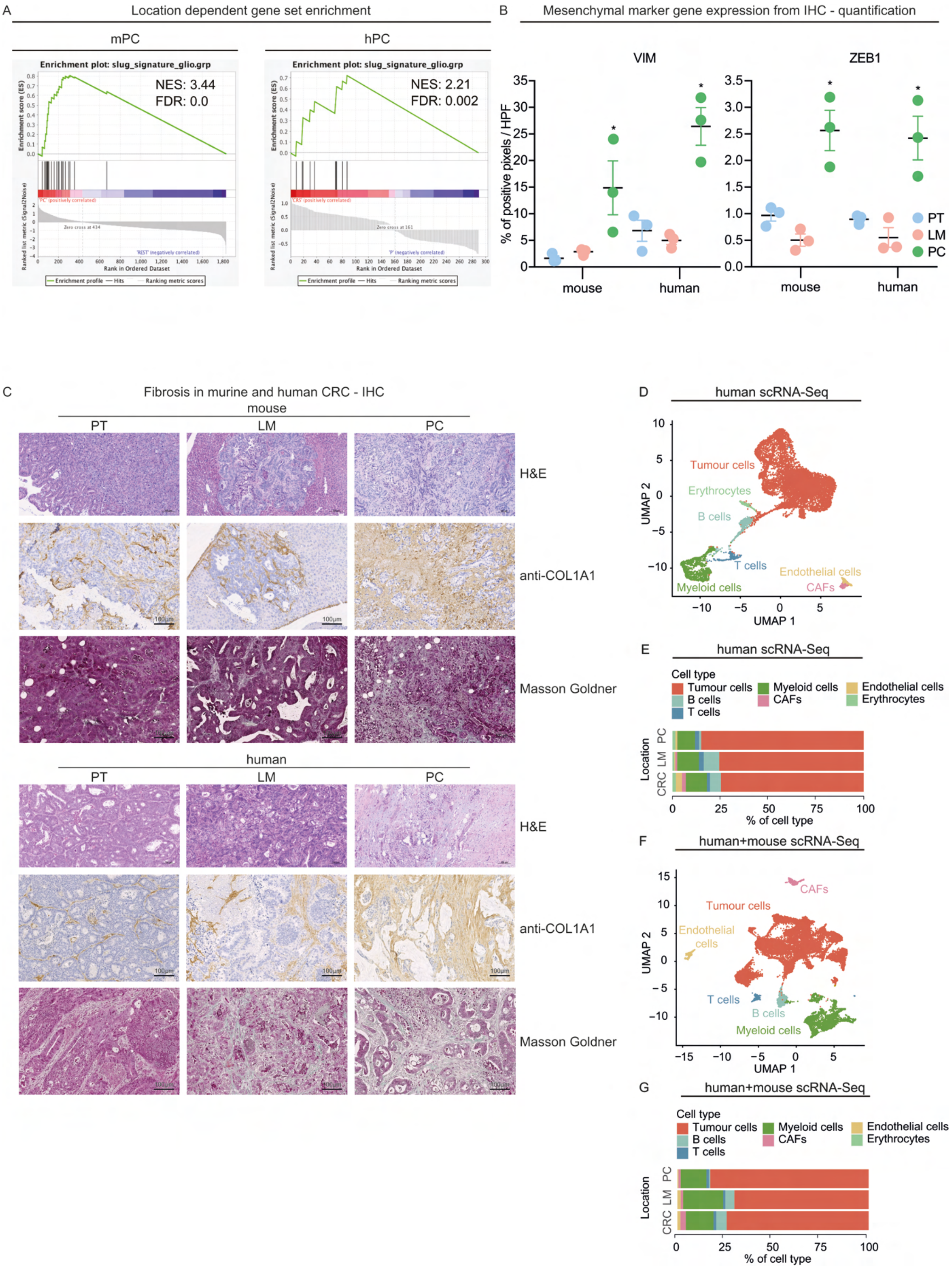
A
Location dependent gene set enrichment
mPC
hPC
Enrichment plot: slug_signature_glio.grp
NES: 3.44
FDR: 0.0
NES: 2.21
FDR: 0.002
B
Mesenchymal marker gene expression from IHC - quantification
VIM
ZEB1
% of positive pixels / HPF
mouse
human
PT
LM
PC
C
Fibrosis in murine and human CRC - IHC
mouse
human
PT
LM
PC
H&E
anti-COL1A1
Masson Goldner
D
human scRNA-Seq
Tumour cells
Erythrocytes
B cells
T cells
Endothelial cells
CAFs
Myeloid cells
UMAP 1
UMAP 2
E
human scRNA-Seq
Cell type
Tumour cells
B cells
T cells
Myeloid cells
CAFs
Endothelial cells
Erythrocytes
CRC LM PC
Location
% of cell type
F
human+mouse scRNA-Seq
G
human+mouse scRNA-Seq

Supplementary Figure 8: Peritoneal carcinomatosis is associated with a mesenchymal phenotype in human and murine CRC

A. GSEA (Signal2Noize) with published Slug gene signature in murine and human PC. NES: normalized expression score. FDR: false discovery rate. **B.** Quantification of VIM and ZEB1 in IHC stainings of murine PT, LM and PC samples per HPF (n=3). * $p<0.05$, Student's T-test. **C.** Representative IHC of murine and human PT, LM and PC for COL1A, as well as Masson Goldner staining. **D.** UMAP plot of human CRC tissue identified by joint application of RCA and CCA and color-coded by cell type. **E.** Proportions of all cell types in human PT, LM and PC on average are shown. **F.** UMAP plot of integrated human and murine CRC identified by joint application of RCA and CCA and color-coded by cell type. **G.** Proportions of all cell types in integrated human and murine PT, LM and PC on average are shown.

A

TME reconstitution by intraperitoneal anti-PD1 therapy in PC

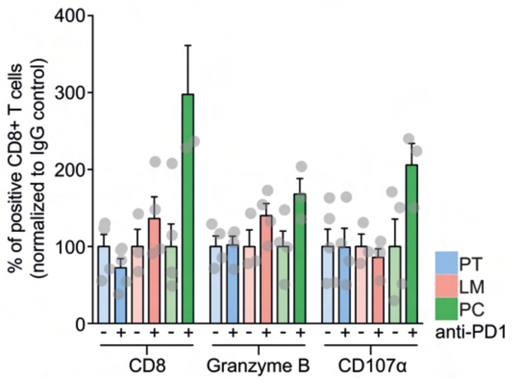

B

Reduction of intraperitoneal tumour mass by intraperitoneal anti-PD1 therapy

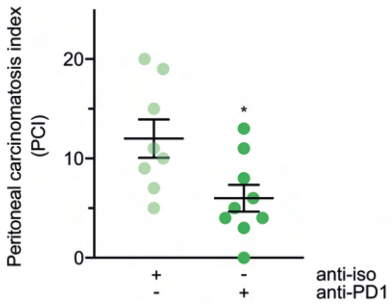

Supplementary Figure 9: Intraperitoneal application of ICB reconstitutes effector T cell function in PC and emphasizes the importance of site-specific CRC therapy

A. Quantification of changes in $CD8^+$ T cell regulation upon intraperitoneal anti-PD1 treatment by FACS. Normalized to IgG control, in %. n=3-5 per group. **B.** Quantification of macroscopic intraperitoneal tumour load (PCI) by diagnostic laparoscopy upon intraperitoneal anti-PD1 treatment (n=8 anti-iso, n=9 anti-PD1). * $p<0.05$, Student's T-test.

Table S1. Western Blot Antibody list.

target	dilution	blocking	company	catalog number
ACTA2	1:1000	5 % BSA	Cell Signaling Technology	#19245S
ACC1	1:1000	5 % BSA	Cell Signaling Technology	#3676
ACTB	1:1000	5 % skim milk powder	Cell Signaling Technology	#4970S
COL1A1	1:1000	5 % BSA	Cell Signaling Technology	#81375S
CDHE	1:1000	5 % BSA	Cell Signaling Technology	#14472S
FAS	1:1000	5 % BSA	Cell Signaling Technology	#3180
LDHA	1:1000	5 % BSA	Cell Signaling Technology	#3582
LOX	1:1000	5 % skim milk powder	Cell Signaling Technology	#58135S
CDHN	1:1000	5 % BSA	Cell Signaling Technology	#13116S
PDHA1	1:1000	5 % BSA	Cell Signaling Technology	#3205
VIM	1:1000	5 % BSA	Cell Signaling Technology	#5741S

ZEB1	1:1000	5 % BSA	Cell Signaling Technology	#70512S

Table S2. IHC Antibody list.

target	dilution	antigen retrieval	company	catalog number
ACTA2	1:320	citrate	Cell Signaling	#19245S
b220	1:100	citrate	eBioscience	#14-0452-82
CD45	1:100	citrate	Cell Signaling	# 70257S
COL1A1	1:100	citrate	Cell Signaling	#81375S
LOX	1:1000	citrate	Genetex	#GTX03201
PAS Kit	-	-	Abcam	#AB150680
PDGFRB	1:150	citrate	Cell Signaling	#3169S
VIM	1:100	citrate	Cell Signaling	#5741S
ZEB1	1:100	citrate	Cell Signaling	#70512S

Table S3. Flow cytometry Antibody list.

target	fluorochrome	company	catalog number
CD45	Alexa Fluor 700	Biolegend	103128
CD3	Brilliant Violet 421	Biolegend	100227
CD3	Brilliant Violet 785	Biolegend	100231
CD3	biotin	Biolegend	100304
CD4	PerCP Cy5.5	Biolegend	100434
CD4	Brilliant Violet 711	Biolegend	100550
CD8	PE Dazzle 594	Biolegend	100762
Foxp3	Alexa Fluor 488	Biolegend	320012
IFNγ	PE/Cy7	Biolegend	505826
IL-17	PE	Biolegend	506933
CD19	Brilliant Violet 650	Biolegend	145517
CD19	biotin	Biolegend	101504
b220	Alexa Fluor 488	Biolegend	103225
b220	PE Dazzle 594	Biolegend	103204
F4/80	Alexa Fluor 488	BioRad	MCA497A488T
CD64	Brilliant Violet 421	Biolegend	139309
CD11b	Brilliant Violet 711	Biolegend	121631
CD11c	Brilliant Violet 785	Biolegend	117336
NK1.1	Brilliant Violet 605	Biolegend	108736
I-A/I-E	Brilliant Violet 510	Biolegend	107636
IL-10	Vio515	Miltenyi Biotec	130-108-097
Gr-1	biotin	Biolegend	108404

Table S4. Gene score list.

apCAF score:

"Cd74", "H2-Aa", "H2-Ab1", "H2-Eb1", "S100a4", "Slpi", "Saa3", "Irf5"

endothelial angiogenesis score:

"Lama4", "Klf6", "Gm1673", "Col14a1", "Hspg2", "Sh3bgrl3", "S100a6", "Col4a2", "Col4a1", "Rhob", "Lgals1", "Pfn1", "Col15a1", "Serpine1", "Htra1", "Sparc"

Endothelial antigen presentation score:

"Cd74", "Egr1", "Cebpd", "H2-Eb2", "H2-Ea", "Igha", "Cldn5", "Itm2b", "H2-D1", "H2-Q1", "H2-Q2", "H2-Q4", "H2-Q6", "H2-Q7", "H2-Q10", "Enpp2", "Jam2", "Sparcl1", "Zc3hav1", "Sertad1", "Jchain", "Cdkn1a", "H2-T24", "H2-T23", "H2-T22", "Gm11127", "Gm7030", "H2-T10", "Gm8909", "H2-T3", "H2-M10.2", "H2-M10.1", "H2-M10.3", "H2-M10.4", "H2-M11", "H2-M9", "H2-M1", "H2-M10.5", "H2-M10.6", "H2-M3", "H2-M2", "H2-M5", "H2-K1", "Socs3", "Cd320", "Irf1", "Fabp5", "Igfbp2", "Klf4", "Gadd45b"

iCMS2_up:

"Vma21", "Pgrmc1", "Nsdhl", "Gabre", "Lamp2", "Spryd7", "Kpna3", "Tbc1d4", "Rnaseh2b", "Uchl3", "Myc", "Nkap", "Edn1", "Ccdc85b", "Rnf113a1", "Gm4737", "Pak1ip1", "C1galt1c1", "Dab2", "Spata13", "Micu2", "Ndfip2", "Prkdc", "Bcap31", "Commd6", "Mcts1", "Tspan6", "Farp1", "Rpia", "Zdhhc9", "Gm7102", "Ldhb", "Gal", "Rida", "Ipo5", "Rrs1", "Nit2", "Rbmx2", "Hsd17b10", "Timm8a2", "Timm8a1", "Ubac2", "Gnpda1", "Gla", "Ftl1-ps1", "Utp14a", "Timp3", "Pdpk1", "Sox4", "Naa10", "Laptm4b", "Slc7a6", "Hibadh", "Serpine2", "Vbp1", "Nek3", "Utp4", "Lage3", "Mrpl13", "Cops5", "Prelid3b", "Sinhcaf", "Rrp9", "Utp23", "Nip7",

"AY761185", "Defa21", "Defa23", "Defa35", "Defa25", "Defa38", "Defa30", "Defa22", "Defa31", "Defa41", "Defa40", "Defa3", "Defa5", "Defa27", "Defa29", "Defa2", "Defa33", "Defa36", "Defa42", "Defa20", "Defa32", "Defa37", "Defa28", "Defa43", "Defa26", "Defa17", "Defa34", "Defa39", "Defa24", "Gtf2f2", "Eif3h", "Rubcnl", "Fundc2", "Pqbp1", "Utp14b", "Cdc16", "C2", "Tsc22d1", "Med30", "Arl4a", "Exosc5", "Proser1", "Sucla2", "Eif3e", "Dkc1", "Neu1", "Upf3a", "Ddah2", "Dnajc15", "Zc3h13", "Cyp2s1", "Immp2l", "Capza2", "Trap1", "Dcaf13", "Atp5e", "Cyb5b", "Pcid2", "Bop1", "Slc52a2", "Cdk4", "Slc3a2", "Top1mt", "Nhlrc3", "Timm17b", "Malsu1", "Krt23", "Fmr1", "Ccl20", "1110038F14Rik", "Emd", "Med4", "Txlng", "Prpf6", "Strap", "Tcf7", "Pan3", "Irs2", "Plin2", "Arfgap1", "Uckl1", "Cdk8", "Mrps31", "Bcl2l1", "Aspscr1", "Plagl2", "Tpd52l2", "Nudt4", "Avl9", "Fnta", "Rpusd1", "Rnf113a2", "Fbl", "Cars2", "Dnajc5", "Scand1", "Nae1", "Phf20", "Cdk5rap1", "Ereg", "Igbp1", "Prox1", "Rab20", "Nkd1", "Qprt", "Pdrg1", "Gpsm2", "Zfp503", "Osbpl2", "Eepd1", "Pofut1", "Mplkip", "Zfand1", "Mapre1", "Asxl1", "Rnf170", "Gm45692", "Chmp4c", "Fggy", "Uchl4", "Helz2", "Supt20", "Arfrp1", "Ogfr", "Prr15", "Slc38a5", "Tpx2", "Rnf6", "Ergic3", "Epb41l5", "Trib3", "Dido1", "Ythdf1", "Ptpro", "Rp9", "Edem2", "Mrgbp", "Ahcy", "Cpne1", "H13", "Las1l", "Nfs1", "Slc20a2", "Epb41l1", "Abracl", "Psma7", "Gtf3a", "Scarb1", "Eif6", "Dpep1", "Armc1", "Rbm39", "Prss23", "Commd7", "Adrm1", "Gm9774", "Pigu", "Csnk2a2", "Dynlrb1", "Cxcl14", "Ctsh", "Gid8", "Trpc4ap", "Golga7", "Slc29a1", "Tdgf1", "Areg", "Rnf114", "Ctsa", "Snrpb2", "Pet117", "Chchd7", "Rab5if", "Rab22a", "Naa20", "Ano9", "Smim26", "Tomm34", "Ftl1", "Rpn2", "Slc5a6", "Psph", "Rtf2", "Cse1l", "Hus1", "Fermt1", "Stx16", "Rb1cc1", "Lypla1",

"Chd6", "Fn1", "Ctnnbl1", "Ascl2", "Aurka", "Uba2", "Nelfcd", "Slc2a8", "Stk4", "Zmynd8", "Serinc3", "Rae1", "Cct6a", "Atp6v1h", "Gm20716", "Dpm1", "Stau1", "Txndc9", "Pcif1", "Gm27027", "Alkbh3", "Snrpb", "Cyp2w1", "Acot8", "Pmepa1", "Ccdc34", "Cebpb", "Atp9a", "Ggh", "Abhd12", "Cox19", "Arhgap45", "1700037H04Rik", "Plcb4", "Rnf43", "Pcmtd2", "Gtf2ird1", "Axin2", "Xrn2", "Slc35c2", "Tbrg4", "Ddx27", "Cdc25b", "Eif2s2", "Rprd1b", "Ncoa3", "Ifitm6", "Gpcpd1", "Pdp1", "Pard6b", "Ndrg3", "Dnttip1", "Pepd", "Vapb", "Mrps17", "Arid3a", "Esf1", "Ift52", "Blcap", "Ywhab", "Mrps24", "Trp53rka", "Trp53rkb", "Pfdn4", "Prap1", "Nxt1", "Ube2c", "Ralgapb", "Oser1", "Pltp", "Tnnc2", "Pex16", "Raly"

iCMS3_up:

"Egln3", "Lpin1", "Try4", "Try5", "Try10", "Prss3", "Gm5771", "Gm10334", "Prss1", "Prss2", "Dpysl2", "Tymp", "Bnip3l", "9530003J23Rik", "F3", "Clu", "Ctnnbip1", "Fut8", "Col16a1", "Atl3", "Me1", "Krt7", "Lipg", "Myof", "Pfkp", "Klk11", "Mmp7", "Cdc42ep1", "Tm4sf4", "Dusp4", "Sytl1", "Tff1", "Sez6l2", "Depp1", "Rab15", "Adam9", "Ndufa4l2", "Tff2", "Rasl11a", "Ramp1", "Eps8l1", "Agrn", "Ppp6r1", "S100a13", "Anxa1", "Stim2", "Kdelr3", "Atp1b1", "Rgs10", "Trim29", "Cd55b", "Cd55", "Pmp22", "Sdr16c5", "Muc5ac", "Dnajc10", "Rgs2", "Aqp3", "Tmem92", "Serpinb5", "Tpgs1", "Car9", "Cfd", "Tnfrsf26", "Tnfrsf22", "Tnfrsf23", "Muc3"

Supplementary Materials

Supplementary Figure 1: A distinct cellular and functional landscape of murine primary CRC, liver metastases and peritoneal carcinomatosis

Supplementary Figure 2: Location-specific metabolic reprogramming of the STEM compartment in multivisceral CRC

Supplementary Figure 3: Inter-metastatic differences in stromal cell dynamics during CRC metastasis

Supplementary Figure 4: Location-specific anti-tumoural immunity during CRC metastasis

Supplementary Figure 5: Impairment of B cell networks in metastasized CRC

Supplementary Figure 6: Inter-metastatic alterations of adaptive immune responses multivisceral CRC

Supplementary Figure 7: Murine multivisceral APTKA CRC mimics human stage IV CRC

Supplementary Figure 8: Peritoneal carcinomatosis is associated with a mesenchymal phenotype in human and murine CRC

Supplementary Figure 9: Intraperitoneal application of ICB reconstitutes effector T cell function in PC and emphasizes the importance of site-specific CRC therapy

Table S1. Western Blot Antibody list.

Table S2. IHC Antibody list.

Table S3. Flow cytometry Antibody list.

Table S4. Gene score list.

Movie S1: Orthotopic surgical transplantation of APTKA organoid cells under the serosa of the murine cecum.

Movie S2: Diagnostic laparoscopy eight weeks after orthotopic surgical transplantation of APTKA organoid cells depicting the development of primary tumours, liver metastases and peritoneal carcinomatosis.
