# Supplementary material for: Single-cell deconvolution reveals high lineage- and location-dependent heterogeneity in mesenchymal multivisceral stage 4 colorectal cancer
Source: J Clin Invest. 2023 Dec 28;134(5):e169576. doi: 10.1172/JCI169576 (PMC10904044; doi:10.1172/JCI169576)

Raw WB scans Berlin et al. for reviewer‘s insight.
All scans are unedited scans of the respective gels. Membranes were cut prior to AB incubation.

Fig. 1I

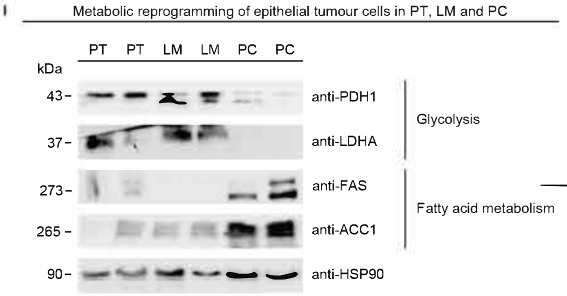

Fig. 2C

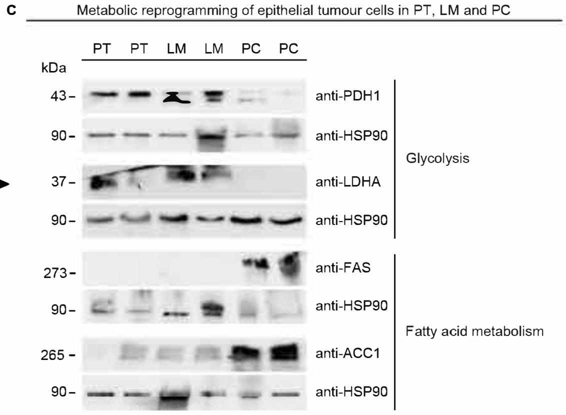

anti-PDH1

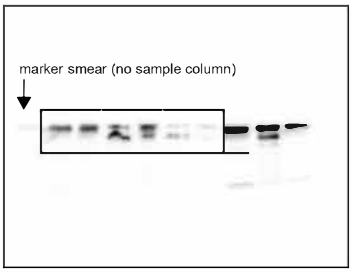

Corresponding loading controls

anti-HSP90

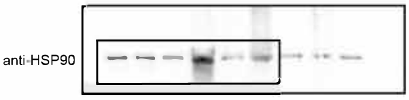

anti-LDHA

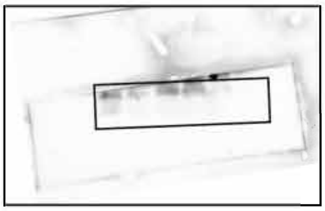

anti-HSP90

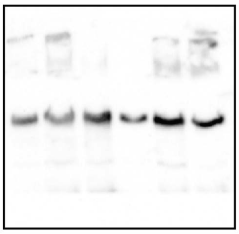

anti-HSP90

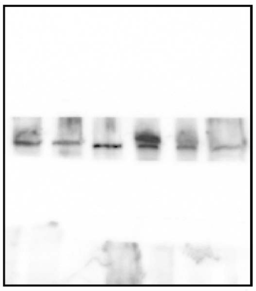

anti-FAS

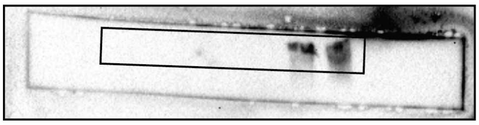

anti-ACC1

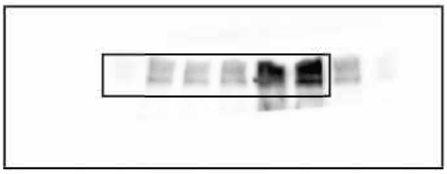

anti-HSP90

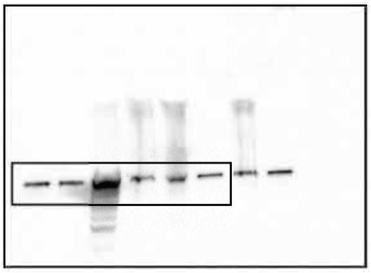

Fig. 8C

Fig. 5H

anti-ZEB1

anti-VIM

anti-N-Cadherin

anti-LOX

anti-β-Actin

anti-α-SMA

COL1A1

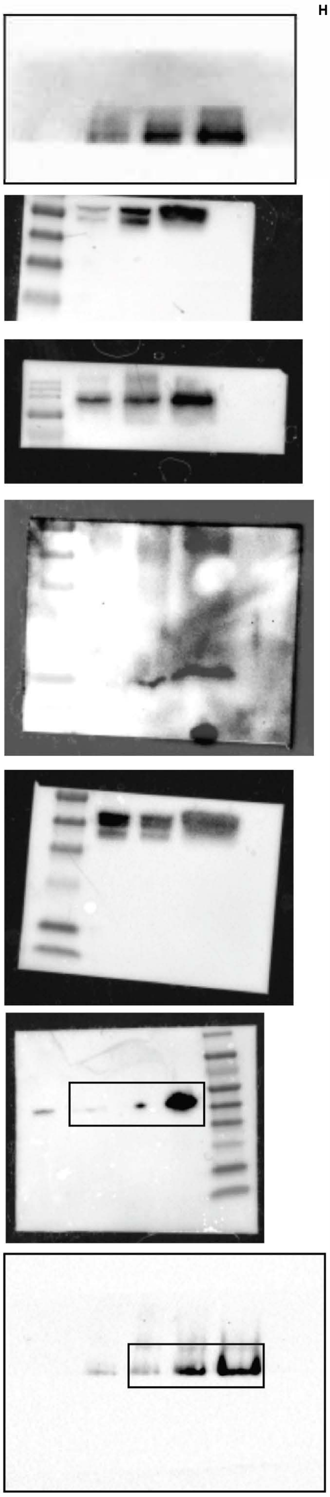

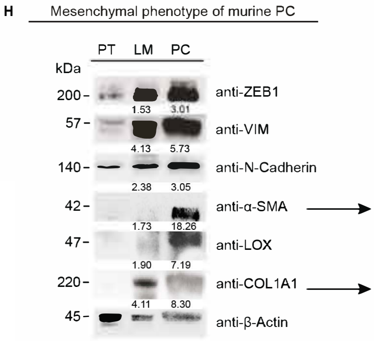

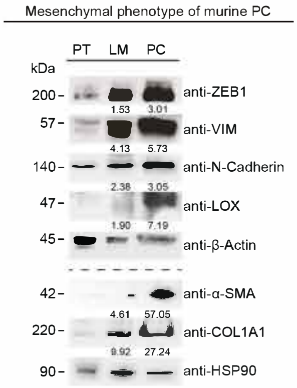

anti-HSP90

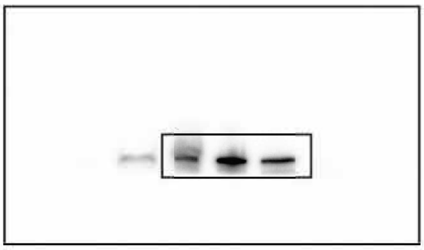

Fig. 5I

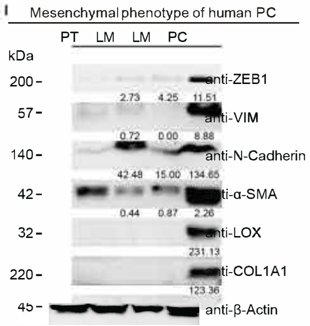

Fig. 8D

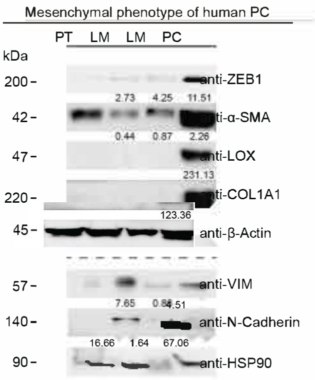

anti-ZEB1

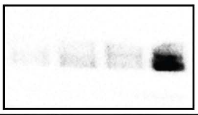

anti-α-SMA

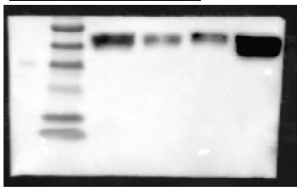

anti-LOX

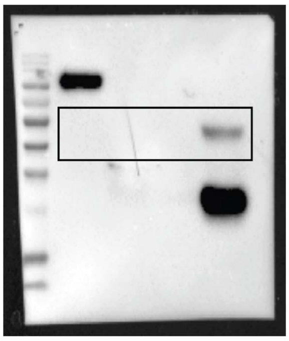

COL1A1

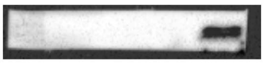

anti-β-Actin

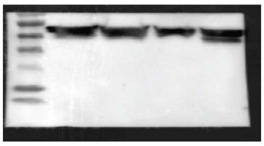

anti-VIM

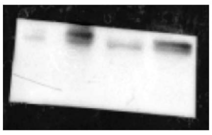

anti-N-Cadherin

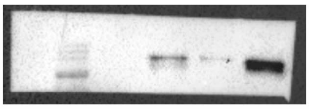

loading control

anti-HSP90

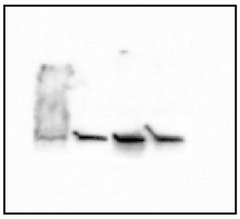

Supplement: Unedited blot and gel images [file jci-134-169576-s025.pdf]
